# Supplementary material for: ‘Relieved to be seen’—patient and carer experiences of psychosocial assessment in the emergency department following self-harm: qualitative analysis of 102 free-text survey responses
Source: BMJ Open. 2021 May 23;11(5):e044434. doi: 10.1136/bmjopen-2020-044434 (PMC8149433; doi:10.1136/bmjopen-2020-044434)
Supplement: Supplementary data [file bmjopen-2020-044434supp001.pdf]

Appendix 1. Additional methodological details

Recruitment

This study was conducted as part of a larger programme of applied research investigating psychosocial assessments and psychological therapies following self-harm in the NIHR Greater Manchester Patient Safety Translational Research Centre. The survey was accessed through a University of Manchester website that contained a summary of the research process, a link to the participants' information sheet, consent form, and post-assessment debrief statement. We expected to recruit approximately 100 respondents, an estimate that was based on our previous studies that were conducted using online surveys.[2-3]

Online survey procedure

The study website, online survey tool, question format (e.g., yes/no, free-text; how the questions were asked), and questions were devised collaboratively with our patient/carer advisory panel via a series of workshops. We included open and closed text questions (e.g., demographics, selecting choices for services; binary response experiences) to elicit participant's views and experiences of assessments and follow-up care (see Box 1). We also asked for self-reported diagnosis, age, gender, education, and where the geographic area where the person resided. Survey questions for patients and carers were similar. Carers were invited to provide proxy information for diagnosis, living arrangements, and employment; and share their views of assessments for the person presenting with self-harm. Participants could tick boxes, skip questions, and/or complete free text responses without word limits. All participants provided informed consent for their participation. Due to the sensitive nature of the topic, links were provided to several online support sites after the assessment. Informed consent was obtained from all participants which was indicated by clicking continue on the survey.

Quinlivan LM, *et al. BMJ Open* 2021; 11:e044434. doi: 10.1136/bmjopen-2020-044434

Box 1. Survey questions

- What is your experience of mental health assessments after you have presented to the emergency department with self-harm? Please tell us what worked well. Please tell us what worked less well.
- How did you feel about having the mental health assessment? How did you feel during the assessment? How did you feel after the assessment?
- Can you think of any reasons when you would be more likely to receive an assessment in the emergency department after self-harm?
- Can you think of any reasons when you would be less likely to receive an assessment following self-harm?
- Have you ever not received or been refused a mental health assessment following self-harm?
- Have you ever refused a mental health assessment?
- Have you ever left the emergency department before being assessed by a mental health clinician?
- Is there anything that would make it more likely that you would accept or stay for a mental health assessment?
- How would you change or do things differently?

Data were analysed and interpreted by a multidisciplinary team that included expertise in psychiatry, mental health services research, psychology, and qualitative methodologies. The public contributors involved in the analyses included people with lived experience of self-harm and attending mental health services after harming themselves, as well as carers of these individuals.

Participant demographics

The majority of patients were residing in England (99/102, 97.1%) and were of White British or Irish ethnicity (92/102, 91.1%). Most patients were either in paid or voluntary work (41/102, 41.4%), or were receiving statutory disability benefits (39/102, 39.4%); 9/102 (8.8%) were students and the remainder were not working (10/102, 10.1%). Many patients had a self-reported psychiatric diagnosis

including the following disorders (that are not mutually exclusive): depression (60/102, 58.8%), emotionally unstable personality disorder (43/102, 42.2%), anxiety disorders (37/102, 36.3%), post-traumatic stress-disorder (25/102, 24.5%), eating disorders (13/102, 12.7%), psychotic disorders (10/102, 9.8%), bipolar disorder (10/102, 9.8%), autism spectrum disorder (7/102, 6.9%), obsessive compulsive disorder (6/102, 5.9%) dissociative disorders (4/102, 3.9%), and dysmorphic disorders (1/102, 1.0%).

References

1. Littlewood, D. L., Quinlivan, L., Steeg, S., Bennett, C., Bickley, H., Rodway, C., ... & Kapur, N. (2019). Evaluating the impact of patient and carer involvement in suicide and self-harm research: A mixed-methods, longitudinal study protocol. *Health Expectations*, 00:1-7.
2. Quinlivan, L., Nowland, R., Steeg, S., Cooper, J., Meehan, D., Godfrey, J., ... & Allen, N. (2019). Advance decisions to refuse treatment and suicidal behaviour in emergency care: ‘it's very much a step into the unknown’. *BJPsych open*, *5*(4).
3. Flynn, S., Raphael, J., Graney, J., Nyathi, T., Williams, A., Kapur, N., ... & Shaw, J. (2019). The personality disorder patient pathway: Service user and clinical perspectives. *Personality and mental health*, *13*(3), 134-143.
