# Supplementary material for: ‘Relieved to be seen’—patient and carer experiences of psychosocial assessment in the emergency department following self-harm: qualitative analysis of 102 free-text survey responses
Source: BMJ Open. 2021 May 23;11(5):e044434. doi: 10.1136/bmjopen-2020-044434 (PMC8149433; doi:10.1136/bmjopen-2020-044434)
Supplement: Author's manuscript [file bmjopen-2020-044434.draft_revisions.pdf]

BMJ Open

BMJ Open is committed to open peer review. As part of this commitment we make the peer review history of every article we publish publicly available.

When an article is published we post the peer reviewers' comments and the authors' responses online. We also post the versions of the paper that were used during peer review. These are the versions that the peer review comments apply to.

The versions of the paper that follow are the versions that were submitted during the peer review process. They are not the versions of record or the final published versions. They should not be cited or distributed as the published version of this manuscript.

BMJ Open is an open access journal and the full, final, typeset and author-corrected version of record of the manuscript is available on our site with no access controls, subscription charges or pay-per-view fees (http://bmjopen.bmj.com).

If you have any questions on BMJ Open's open peer review process please email info.bmjopen@bmj.com

BMJ Open

"Relieved to be seen" - patient and carer experiences of mental health assessment in the emergency department following self-harm: qualitative analysis of 102 free-text survey responses

Journal:	*BMJ Open*
Manuscript ID	bmjopen-2020-044434
Article Type:	Original research
Date Submitted by the Author:	03-Sep-2020
Complete List of Authors:	Quinlivan, Leah; The University of Manchester, Biology, Medicine, and Health<br>Gorman, Louise<br>Littlewood, Donna ; The University of Manchester,<br>Monaghan, Elizabeth<br>Barlow , Steven<br>Campbell, Stephen; The University of Manchester, Centre for Primary Care<br>Webb, Roger; The University of Manchester, School of Community Based Medicine<br>Kapur, Navneet; The University of Manchester, Centre for suicide prevention
Keywords:	Suicide & self-harm < PSYCHIATRY, Quality in health care < HEALTH SERVICES ADMINISTRATION & MANAGEMENT, PSYCHIATRY

SCHOLARONE™
Manuscripts

I, the Submitting Author has the right to grant and does grant on behalf of all authors of the Work (as defined in the below author licence), an exclusive licence and/or a non-exclusive licence for contributions from authors who are: i) UK Crown employees; ii) where BMJ has agreed a CC-BY licence shall apply, and/or iii) in accordance with the terms applicable for US Federal Government officers or employees acting as part of their official duties; on a worldwide, perpetual, irrevocable, royalty-free basis to BMJ Publishing Group Ltd ("BMJ") its licensees and where the relevant Journal is co-owned by BMJ to the co-owners of the Journal, to publish the Work in this journal and any other BMJ products and to exploit all rights, as set out in our licence.

The Submitting Author accepts and understands that any supply made under these terms is made by BMJ to the Submitting Author unless you are acting as an employee on behalf of your employer or a postgraduate student of an affiliated institution which is paying any applicable article publishing charge ("APC") for Open Access articles. Where the Submitting Author wishes to make the Work available on an Open Access basis (and intends to pay the relevant APC), the terms of reuse of such Open Access shall be governed by a Creative Commons licence – details of these licences and which Creative Commons licence will apply to this Work are set out in our licence referred to above.

Other than as permitted in any relevant BMJ Author's Self Archiving Policies, I confirm this Work has not been accepted for publication elsewhere, is not being considered for publication elsewhere and does not duplicate material already published. I confirm all authors consent to publication of this Work and authorise the granting of this licence.

For peer review only

Title: "Relieved to be seen" - patient and carer experiences of mental health assessment in the emergency department following self-harm: qualitative analysis of 102 free-text survey responses

Author details
Leah Quinlivan, PhD[1,2], Research Fellow; Louise Gorman, PhD[1], Research Associate; Donna Littlewood, PhD[1] Research Associate; Elizabeth Monaghan, Public contributor; Stephen J Barlow, Public Contributor; Stephen Campbell[3], Professor of Primary Care Research; Roger T. Webb, Professor of Mental Health Epidemiology;[1,2,3] Prof Nav Kapur, FRCPsych[1,2,3] Professor of Psychiatry and Population Health and Honorary Consultant Psychiatrist.

1. Centre for Mental Health and Safety, University of Manchester, England
2. Manchester Academic Health Science Centre, University of Manchester, England
3. NIHR Greater Manchester Patient Safety Translational Research Centre, The University of Manchester, Manchester academic Health Science Centre, Manchester, UK.
4. Greater Manchester Mental Health NHS Foundation Trust, Manchester, England

Corresponding author: Dr Leah Quinlivan
Address: Jean McFarlane Building, University of Manchester, Oxford Road, Manchester M13 9PL
Telephone: +44 (0)161 275 0727
Email: leah.quinlivan@manchester.ac.uk

Word count: 3984
Tables: 4
Figures: 1
References: 25 excluding 5 technical references
Appendices: 2

For peer review only

ABSTRACT

Objectives: We sought to explore patient and carer experiences of psychosocial assessments following presentations to hospital after self-harm.

Design: Qualitative analysis of free-text responses to an open-ended online survey.

Setting: Between March and November 2019, we recruited 102 patients and carers aged 18 or over from 16 English mental health trusts, community organisations, and via social media.

Results: Psychosocial assessments were experienced as helpful on some occasions but harmful on others. Participants' felt better, less suicidal, and less likely to repeat self-harm after good quality compassionate and supportive assessments. However, negative experiences during the assessment pathway were common and, in some cases, contributed to greater distress, less engagement, and further self-harm. Participants reported receiving negative and stigmatising comments about their injuries. Others reported that they were refused medical care or an anaesthetic. Stigmatising attitudes from some mental health staff centred on self-harm as a maladaptive behavioural issue, service use, and diagnosis.

Conclusion
Our findings highlight important patient experiences that can inform service provision and they demonstrate the value of involving patients/carers throughout the research process. Psychosocial assessments can be beneficial when empathetic and collaborative but less helpful when overly standardised, lacking in compassion, and waiting-times are unduly long. Patient views are essential to inform practice, particularly given the rapidly changing service context during and after the Covid-19 emergency.

Keywords: self-harm; suicide risk; liasion psychiatry; psychosocial assessments; qualitative research

For peer review only - http://bmjopen.bmj.com/site/about/guidelines.xhtml

Strengths and limitations

- Understanding what works and does not work for patients when receiving psychosocial assessments following self-harm is key to improving practice, however such evidence is limited.
- This is the largest qualitative study on psychosocial assessments following self-harm and the only study to have also included carer perspectives.
- Our extensive patient and carer involvement and use of a qualitative survey enabled us to access a marginalised and stigmatised group of patients and carers with substantive unmet healthcare needs.
- A limitation of this study is the use of a non-probability survey design. However, our aim was to provide qualitative experiential data and not to generalise to the broader population.
- The respondents may differ from other patients/carers, who did not see or respond to the survey in important ways such as help-seeking and healthcare needs.

For peer review only

Statements:

Funding statement
This work was funded by the National Institute for Health Research (NIHR) Greater Manchester Patient Safety Translational Research Centre. The views expressed are those of the authors(s) and not necessarily those of the NIHR or the Department of Health and Social Care.

Competing interests
NK is a member of the Department of Health's (England) National Suicide Prevention Advisory Group. He chaired the NICE guideline development group for the longer-term management of self-harm and the NICE Topic Expert Group (which developed the quality standards for self-harm services). NK is currently chair of the updated NICE guideline for Depression and Topic Advisor to the new NICE self-harm guideline. He is also supported by the Greater Manchester Mental Health NHS Foundation Trust.

Acknowledgements:
We wish to thank the participants who have taken the time to generously share their often-painful experiences, and also our patient and carer panel for their involvement and insights throughout the process. We are also grateful to our GM NIHR PSTRC staff stakeholder panel, and to the FAST-R panel for their input into the study. Finally, we wish to thanks staff at the mental health trusts and clinical research networks, as well as community organisations for facilitating the research and assisting with the recruitment.

Authorship & contribution
All authors made substantial contributions to the study. LQ, DL, RW, and NK designed the study with input from our patient, carer, and staff stakeholder panels. LQ coordinated data collection with assistance from DL. LQ and LG did the analyses with input from the patient and carer advisory panel, in addition to: LM, SB, SB, LM, DL, RW, and NK. LQ interpreted the results with input from LG, LM, SB, DL, RW, NK, and LQ wrote the first draft. All authors contributed to subsequent drafts and approved the final version. All authors take responsibility for the integrity of the data and accuracy of the data analysis. NK is the guarantor of the study.

Data availability
The data that support the findings of this study are not publicly available due to restrictions of the research (consent and information that could compromise the privacy of some research participants).

Patient and public involvement
Our patient and carer advisory panel were involved all aspects of the research process including the design, conduct, reporting, and dissemination plans for the research. Two panel members (EM, SJB) with lived experience in this area contributed in-depth to the analyses and are co-authors of this paper. This research was also reviewed by a team with experience of mental health problems and their carers who have been specially trained to advise on research proposals and documentation through the Feasibility and Acceptability Support Team for Researchers (FAST-R): a free, confidential service in England provided by the National Institute for Health Research (NIHR) funded Maudsley Biomedical Research Centre via King's College London and South London and Maudsley NHS Foundation Trust. There was patient and public involvement input into our dissemination plan, which includes communicating key findings to relevant patient groups, carers, and mental health services.

For peer review only - http://bmjopen.bmj.com/site/about/guidelines.xhtml

INTRODUCTION

Self-harm is a common antecedent and strong risk factor for suicide.[1-3] Repeat self-harm occurs frequently and people who harm themselves more than once have an even higher risk of suicide.[3] Although hospital presentations represent the 'tip of the iceberg' for self-harm,[4] they provide an important opportunity for intervention via the provision of good quality care.[5] Liaison psychiatry services are an integral part of the self-harm care pathway.[2] Specialist teams are typically situated in acute hospitals and provide liaison care for patients on wards and in the emergency department.[2] Psychosocial assessments are a core component of care and are recommended for all patients presenting to hospital services having harmed themselves.[2] Good quality assessments may help to prevent repeat self-harm. [6-7]

Liaison psychiatry has rapidly transformed to manage the consequences of the covid-19 pandemic on service provision.[8] Mental health services are in flux and long-term changes are likely.[8] However, psychosocial assessments remain a core part of practice.[8] To ensure high quality care in liaison psychiatry, an enhanced understanding from the perspective of patients and carers as to what helps and does not help is now needed more than ever.[9]

Several studies of variable methodological quality and reporting standard have investigated healthcare service experiences following self-harm.[10] However, only two relatively small and geographically local studies have focussed on psychosocial assessments,[11-12] and none have included carer perspectives or involved patients and carers throughout the research process.

Between March and November 2019, we collaborated with mental health trusts in England and other community organisations to conduct a qualitative online patient and carer survey on psychosocial assessments in relation to self-harm. For the current study, the specific objective was to explore positive and negative patient experiences of psychosocial assessments in the emergency department in a large sample of patients and carers.

For peer review only - http://bmjopen.bmj.com/site/about/guidelines.xhtml

METHODS

Ethics statement

The authors assert that all procedures contributing to this work comply with the ethical standards of the relevant national and institutional committees on human experimentation and with the Helsinki Declaration of 1975, as revised in 2008. All procedures involving human subjects/patients were approved by [Greater Manchester Central Research Ethics Committee (REC No: 18/NW/0839)].

Design and sample

Using a pragmatist epistemological approach, we conducted a qualitative online survey[13] to investigate patient and carer experiences of assessments following self-harm. Our aim was not to generalise to a wider population or to estimate prevalence, but to instead provide qualitative experiential data about the assessment process.

Recruitment

We invited patients and carers aged 18 or over with experience of self-harm (defined as intentional self-poisoning or self-injury irrespective of suicidal intent[2], mental health assessments, and psychological therapies to participate in a national online survey. We did not include people under the age of 18 because of differences in service provision for child and adolescent mental health services (CAMHS) in England.[14] To capture a wide range of experiences, we recruited participants through 16 NHS mental health trusts around England, social media (e.g., Twitter, Facebook), community organisations (e.g., charities, patient groups), and newsletters, from April 2019 through November 2019. We closed recruitment when there was a sufficient rich and range of responses for the free-text questions and due to pragmatic constraints (e.g., study deadlines). The recruitment flow chart and additional methodological information are presented in the Appendices.

For peer review only

Analysis

We used thematic analysis to explore patterns across the dataset that represented participants' experiences.[13] Responses to structured questions were analysed using deductive methods and inductive methods were used to capture additional codes and context across the data set.[13,15] Our patient and carer advisory group coded the data and developed the coding framework, which was applied across the dataset. After immersion and familiarisation with the data, LG and LQ independently coded the full study dataset with this framework and reviewed convergence for codes and themes. Throughout the iterative coding process, the researchers reviewed and revised the newly developed inductive codes and the application of the coding framework. Working together with patients and carers, themes were constructed and revised from group discussion to enhance their clarity and relevance. We analysed responses from subgroups (e.g., patients/carers, gender, age-groups) together because the responses consistently overlapped. The final thematic structure and illustrative quotes were agreed through discussion amongst the team.

Descriptive quantitative analyses were performed using SPSS version 22;[16] NVivo 12 Software [17] was used for data management.

For peer review only

RESULTS

Descriptive quantitative results

In total, 102 participants provided text responses on experiences of psychosocial assessments in the online survey. The majority of participants were patients (88/102, 86.3%), and the remainder were carers (14/102, 13.7%). Patients were aged between 18 and 75 and their median age was 34. Carers were aged between 49 and 59 and their median age was 56. Most patient (72/88, 81.8%) and carer (13/14, 92.9%) respondents were women.

For peer review only

Qualitative results

Experiences of psychosocial assessments

Psychosocial assessments are embedded in pathways of care when patients attend the emergency department after harming themselves. Participant experiences reflected the temporal and dynamic nature of presentations for self-harm (e.g., the assessment process may take place over several hours). In order to understand what helps and does not help for assessments, it was important that we considered the context of what went before and came after the assessment. To capture the context of psychosocial assessments, the themes are presented under stages of the care: (1) before the assessment; (2) during the assessment; and (3) after the assessment. Figure 1 presents the themes and subthemes throughout the process. Additional supporting quotations are in Tables 1, 2, and 3.

For peer review only - http://bmjopen.bmj.com/site/about/guidelines.xhtml

[INSERT FIGURE 1 HERE]

Figure 1. Themes and subthemes across the three assessment process stages (1. Before the assessment; 2. During the assessment; 3. After the assessment)

For peer review only

Themes

Stage 1. Before the psychosocial assessment: What helped?

Empathetic, supportive, and humanising care

Positive experiences of the pre-assessment process centred on empathetic, supportive emergency department staff who reassured participants and humanised the experience. Many people were anxious and uncertain about the waiting times and assessment process. Clear communication, check-ins from staff, and empathy helped to reassure and encourage people to stay for the assessment. Having support during the initial stages helped some participants feel "looked after" (R94, Patient), which was helpful when they were distressed. Soothing aids such as heat packs and/or drinks and emotional support helped to humanise the process while participants waited for the assessment.

What did not help?

Waiting times/medically cleared

Most participants reported long and frustrating waiting times for their mental health assessment. There was little communication about time-frames, which left people unsure of what was happening, when they would be seen, and by whom, etc. Often it was not possible to speak to a mental health professional until the physical injury had been treated (medically cleared), which added further delays, distress, and frustration. Some participants were left alone for long periods of time whist they waited to see a mental health clinician leading to greater uncertainty about the process.

Emergency department environments

Some people were offered a separate waiting room and support whilst they waited for the assessment which was helpful. However, for others the emergency department environment felt physically and psychologically unsafe. The noise, intensity, and lack of privacy increased their distress, particularly where there was a lack of communication over the process and waiting times. Others waited in physically unsafe rooms for long periods of time. One patient reported harming herself with "sharp objects" left in a bin whilst she waited over five hours to see mental health five hours to see mental

For peer review only - http://bmjopen.bmj.com/site/about/guidelines.xhtml

health practitioners (R01, Patient). One mother reported that her son harmed himself and required stitches in an unsafe "side room without scrutiny or supervision" (R100, Carer).

Stigmatising attitudes and poor medical care

Participants reported a lack of parity of esteem in the emergency department between self-harm and physical healthcare. Negative interactions with general medical staff while waiting for an assessment or during medical treatment for self-harm were reported as common. Some felt they had been treated with contempt and as a lower priority than those with physical health issues, especially during busy periods. Others reported receiving punitive medical care from some general medical staff which included insensitive and derogatory comments about their injuries. Some participants reported that they were refused pain-relief because they had harmed themselves: "You never had an anaesthetic when you cut so we won't give one now to suture your wounds!" (R08, Patient). Others were refused medical treatment because it was felt that they would repeat self-harm: "I was told that it would be a waste of time because 'you'll just go and do it again, or cut through our stitches'") (R17, Patient), leaving some people at risk of further physical health complications or infection.

Coercion and control

Some participants attended the emergency department because of family or the police. Coercive practice from staff whilst waiting for the assessment compounded their lack of control in the process. Some were threatened with police involvement or a criminal record if they did not comply with assessments, which served to heighten feelings of disempowerment: "I don't have a choice. They decide if I need it and need to stay or they'll call the police" (R105, Patient). This was evident where security personnel of a different gender to the patient were present during toilet breaks, further exacerbating feelings of disempowerment. The use of coercion in the emergency department adversely affected some participants' engagement with the assessment process. One person described how they were more likely to refuse an assessment: "because of coercion and threat of police being called if I leave or being sectioned if I don't comply" (R06, Patient). This disempowering approach at

For peer review only - http://bmjopen.bmj.com/site/about/guidelines.xhtml

the initial stage of the assessment contributed to an avoidance of future help-seeking for some participants.

Table 1: Before the psychosocial assessment: Themes and exemplar quotations		
**Psychosocial assessment stage**	Themes	Example quotes
**What helped?**	Empathetic, supportive, and humanising care	"I arrived at the emergency department at 2am after a breakdown and had to wait over 5 hours for the mental health team to see me on the day shift. I had self-harmed and told the staff this, and I told them that I was feeling suicidal. I didn't refer myself to the hospital and I wanted to leave during the 5-hour wait but I was encouraged to wait to see the doctor before I could leave. The nurses on shift were pleasant and one- on-one with me a few times, offered drinks, etc." (R86, Patient)
**What did not help?**	Waiting times/medically cleared	"If we had gone into A&E for self-harm, psychiatric liaison would refuse to see my daughter until she has completed her treatment, be it stitches, stapling or NAC. They would refuse to do an assessment until she was medically fit/cleared; we often had to wait 5, 6, 7 hours for someone to do a mental health assessment." (R20, Carer)
	Emergency department environments	"It was unhelpful when there was no transparency about how long I would be waiting to see the mental health team so that I could make an informed choice - worst incident was admitted at 9pm, told it would be about 2 hours to see the team but needed to get the all-clear from the doctors first with regards to physical health. Had to share room with a man who was very intoxicated and who had soiled himself, which was very frightening as a 21-year-old female. Then was moved to corridor for some time, until being moved back to the waiting room once my obs were stable as no more space on the ward. I was not able to see the mental health team until 10am the following day and later found out they had gone home for the night". (R41, Patient)
	Stigmatising attitudes and poor medical care	"in many cases, staff lacked compassion. Such as invalidating my distress, stigmatising responses such as 'wow you really meant to kill yourself, didn't you!!', exclaiming at the severity of my previous scarring and saying I was 'adding to the collection', saying that my pain threshold must be high and deciding not to give me any pain relief or medications when stitching or cleaning wounds (almost as if it was to be a punishment for self-harming), saying that I was 'wasting time' and other people had 'real' injuries". (R17, Patient)
	Coercion and control	"Don't like it when male security escort me to toilet in case I abscond" (R01, Patient).

For peer review only - http://bmjopen.bmj.com/site/about/guidelines.xhtml

Stage 2: During the assessment: What helped?

Collaborative assessments and engaged communication

The way the assessment was conducted made a major difference to the person's experience. Active listening, good communication, and engaging with the person facilitated the collaborative process during the clinical encounter. Assessments were more helpful when the person felt they were given control and choice (collaborative assessments). Participants felt heard and respected when they were included in decision making and understood what was happening in the assessment.

Space and time to talk

Opportunity and time to talk about their distress was helpful for some participants. One participant reported that the assessment was the first time that he had spoken to anyone about his mental health or suicidal ideation (R04, Patient). Other participants found having the space and time to talk helpful irrespective of the follow-up care that they received.

Recognition and reassurance

Compassionate care was a key determinant of a helpful assessment. Many participants experienced internalised stigma, anxiety, and a range of intense emotions during the assessment. Some people felt shame and guilt about presenting to hospital with self-harm, which was exacerbated by poor interactions with some medical staff. Others were nervous about the consequences of the assessment and/or judgement from staff if they disclosed the intensity of their thoughts: "Uncomfortable. It's hard to open up and/or share thoughts others might find concerning/abnormal" (R69, Patient).

Assessments worked well when mental healthcare staff recognised the distress that led to the participant's self-harm and their anxiety over the process. Assessments were helpful for many participants when they were reassured that they did the "right thing in coming to A&E" (R09, Patient). Kindness and consideration helped to establish a therapeutic relationship and alleviate some participants' anxiety during this sensitive time. Compassionate care and active listening from mental health staff helped to humanise the assessment experience and develop a therapeutic relationship.

For peer review only

Sensitivity

Assessments were a vulnerable time and participants valued sensitivity from staff. Asking questions sensitively was important for some participants because talking about past traumatic experiences could be distressing. Assessments were more beneficial when staff acknowledged patient's physical pain and psychological distress during the assessment.

Help

Participants attended the emergency department to seek help after harming themselves. Assessments were evaluated more positively when participants received collaboratively developed safety plans, referrals to promptly accessible specialist services, or provided follow-up phone calls from mental health staff. Actionable outcomes from the assessment helped to legitimise and validate participants' distress and reasons for seeking help.

During the assessments: What did not help?

Generic tick-box assessments

Unhelpful assessments were overly standardised and generic for some participants. Some participants felt that the assessments were designed to fit their psychological distress and complex experiences into "neat little boxes" (R106, Patient). In these instances, the focus was on ticking boxes rather than hearing the person had to say and little room was left to explore and assist the person with their immediate distress in this "bureaucratic and uncaring" process (R86, Patient). The lack of opportunity to meaningfully talk about their distress negated the potential therapeutic value of the assessment for some people.

Intense and invasive questions

Assessments could be intense and intrusive for some people. The combination of overly standardised assessments and a lack of therapeutic engagement made it difficult for some people to talk about their distress. In some cases, the intensity of the assessment left people feeling more vulnerable.

For peer review only - http://bmjopen.bmj.com/site/about/guidelines.xhtml

Unnecessarily repeating details

For others, assessments placed too much emphasis on the past and did not focus on their current level of distress. Many shared their life experiences and the reasons behind their self-harm during previous assessments. Being asked to give this information again exacerbated the lack of continuity of care, assumptions that notes have not been read, and frustrations with the participants' experience of continuously poor understanding of some mental health conditions among some staff.

Stigmatising attitudes during the assessment

Stigmatising attitudes changed slightly across the assessment stage. Misconceptions about self-harm and attention seeking were an issue throughout the process though. However, during the pre-assessment stage, stigmatising attitudes often focused on the act of self-harm and a lack of parity of esteem between self-harm and physical health injuries. Participants reported receiving negative comments about wasting healthcare resources and their injuries. During the assessment stage, stigmatising attitudes commonly focused on psychiatric diagnosis and patterns of service use for many people. Many felt judged during the assessment because of their self-harm, particularly if they had previously presented to services. Some participants described issues that centred around self-harm as a behaviour and that seeking help indicated "attention seeking behaviour." (R08) Perceiving self-harm as a behaviour indicated that the person was at fault for hurting themselves with little regard for any trauma or other psychological determinants. Some participants felt that they were spoken to condescendingly because they were "misbehaving".

Many participants with a diagnosis of personality disorder reported experiencing stigmatising attitudes from staff once they became aware of their diagnosis as if this indicated that they must be "attention seeking" and a "time-waster" when they sought help in the emergency department (R20, Carer; R17, Patient). Some participants reported that the compassion and interest in the person changed during the assessment because of their diagnosis. Many participants felt unsupported and judged because of

For peer review only - http://bmjopen.bmj.com/site/about/guidelines.xhtml

their service use. Assessments were rushed, and their diagnosis negated any possible risk of further self-harm and suicide from the perspective of healthcare staff.

Table 2: During the psychosocial assessment: Themes and exemplar quotations

	Themes	Example quotes
**During the assessment: What helped?**	Collaborative assessments and engaged communication	"In any situation, what works well is when I feel listened to and like I had some input and agreement into the decision and follow up and most importantly that I understood the situation and why it was happening." (R34, Patient)
	Space and time to talk	"I knew I wouldn't get any help but at the same time it was good to have the opportunity to talk to someone." (R36, Patient)
	Recognition and reassurance	"The last two occasions I have had an assessment with a psychiatric-liaison practitioner, they have been really positive. I was made to feel as a human and felt as though how I was feeling was validated… Initially, I was nervous. I find talking openly very difficult and tend to fabricate answers due to being scared of the repercussions. The nurses I saw could sense my apprehensions but were encouraging with me to speak. Afterwards, I felt like I wanted them to be a constant part of my mental health recovery, knowing that the reason why I saw them was due to nearly losing my life. They temporarily restored my faith in the MH system." (R59, Patient)
	Sensitivity	"What has worked well, is staff being sensitive with their questioning about what led up to the self-harm, how they could help my distress, how much physical and mental pain I was in and what I needed, if I needed sensory soothing aids (e.g. heat packs) while they undertook the assessment and asked questions about my self-harm and mental health history. Humanising, person-centred care that is trauma-informed is helpful and works well in making a difficult situation and experience easier to bear." (R17, Patient)
	Help	"I got taken into a separate room to discuss my situation and options available - the staff member listened well and took all my intentions seriously. I felt heard. I was sent home afterwards with a detailed "crisis plan of action" that I could read and share whenever things were getting difficult. This was helpful." (R113, Patient)
**During the assessment: What did not help?**	Generic tick-box assessments	"What didn't work well was being told I would be okay, the nature of a checklist-like set of questions to evaluate someone's mental health left no room for me to really talk about how I was actually feeling." (R09, Patient)
	Intense and invasive questions	"These assessments were often quite intense and invasive, in so much as a professional with whom I would never again have contact sought to dredge up my entire life history, only to send me home feeling more unsettled than when I'd arrived…" (R101, Patient)
	Unnecessarily repeating details	"Sometimes there is little point in repeating the reasons or trying to explain why I have self-harmed. People often don't understand it as it is linked to OCD. I get frustrated when people don't understand and it then makes it difficult to work with professionals." (R95, Patient)

For peer review only - http://bmjopen.bmj.com/site/about/guidelines.xhtml

**During the assessment: What did not help?**	Stigmatising attitudes during the assessment	"What doesn't work is being told I am doing it for attention, and that they know better than me what is helpful, so they won't change the plan. The most unhelpful things are to be told that I didn't really want to kill myself because I'm not dead and that it is up to me if I kill myself." (R116, Patient)<br><br>"When I have presented with a diagnosis of emotionally unstable personality disorder, triage was still quick, but staff have been cold and lacking in empathy and compassion. Assessments were treated, almost with boredom, and I've been discharged despite being a current risk of suicide or further self-harm." (R47, Patient)

Stage 3: After the assessment: Consequences

Hope

Feelings after the assessment were closely related to the perceived quality of the assessment and support received during and afterwards. More positive emotions were reported after collaborative and compassionate assessments that provided practical help from the mental health clinician. Humanising care helped to lessen the person's distress and ease the acuity of their psychological crisis. Emotive challenges of the assessment process were offset for some because they received a first step towards seeking help and managing their self-harm. Humanising and supportive care helped to helped to provide participants with hope.

Harm

Most participants felt they would get help from the assessment but over a third indicated that they were harmed or felt worse by the process. Some reported feeling worse after the assessment because they experienced a lack of therapeutic engagement and support. Abrupt transitions out of the assessment left other people feeling abandoned and discarded. Positive effects of the assessment were negated for some people, with some participants reporting a worsening of their distress.

Negative and frustrating experiences of aftercare and support led many participants to perceive the assessments as a "waste of time" (R128, Patient). Receiving out-of-date signposting information was frustrating for some people and others found the clinician's advice to be overly simplistic. Feeling

For peer review only - http://bmjopen.bmj.com/site/about/guidelines.xhtml

unsupported during and after assessments led to despondency, hopelessness, and increased distress for some participants. In several instances, negative experiences of the assessment process and the lack of support led to further thoughts and acts of self-harm.

Help-seeking and engagement

Experiences of attending the emergency department following self-harm had a cumulative effect and resulted in far-reaching consequences on help-seeking and engagement with mental health services for some participants. Some reported that the lack of a therapeutic assessment, inadequate follow-up care and/or long waiting lists to access support increased their levels of distress and disillusionment. Over a third of participants reported that they were less likely to seek help or engage with assessments and/or were more likely to leave the emergency department without an assessment.

Table 3: After the psychosocial assessment: Themes and exemplar quotations

**Themes**	
**Hope**	"before and during, I felt ashamed, like a fraud and saddened that I was putting my family and friends through all this, and even more so that I was now putting a drain on resources that could off been used for someone else who needed it more than I. After, I felt supported by family with me and the action plan we had all discussed. I felt there was a glimmer of hope in all the darkness." (R113, Patient)
**Harm**	"I felt that there was no room to explore what was actually going on, that I was given little opportunity to express myself, and like the whole process was a bit of a tick-box exercise, rather than a supportive conversation or a way to come up with helpful suggestions. 9 times out of 10 these assessments left me feeling worse." (R101, Patient)<br><br>"The RAID assessment made me feel listened to and gave me a next step. But the next step was pointless. I was back home feeling like nothing changed. So, I felt in many ways worse." (R70, Patient)<br><br>"They tell you to simply go listen to music or have a bath and then discharge you and as a result, you attempt to end your life." (R30, Patient)
**Help seeking and engagement**	"I felt judged whilst being asked questions about how I was feeling, and like I was being misunderstood and like I wasn't being taken seriously. And after the assessment and after discharge from A&E the vague advice I had been given made me even less likely to return to A&E in future." (R09, Patient)<br><br>I didn't feel like I was being treated as a very high priority, and went home feeling like there's no point in going to A&E when I'm suicidal from now on (even though that's what all health professionals seem to suggest)." (R15, Patient)

DISCUSSION

Main findings

We found that the psychosocial assessment process was experienced as helpful on some occasions but harmful on others. Participants reported feeling better, less suicidal, and less likely to repeat self-harm after receiving compassionate care and collaborative assessments that focused on building a therapeutic relationship and providing help. Conversely, the pathway in the emergency department and the manner by which the assessment was conducted may have resulted in iatrogenic harm for some patients. Reports of substandard medical care and derogatory comments were common among participants, and compounded by what appear to be pre-conceived ideas among some staff members regarding service use patterns, self-harm repetition, and perceived attention-seeking behaviour. For some participants, cumulative experiences of long-waiting times, stigmatising attitudes, disjointed care, and overly standardised assessments contributed to heightened distress, disengagement, and in some cases further self-harm episodes.

Strengths and limitations

We conducted an online survey with non-probability sampling to recruit our participants, an approach that is subject to potential selection bias.[18] However, our aim was not to generalise to a broader population but to provide qualitative contextual experiences of attending the emergency department for psychosocial assessments. Self-harm is often stigmatised. Recruiting individuals who have harmed themselves can be challenging, especially for emergency department studies.[10-12,14] Our co-designed qualitative online survey enabled us to investigate a marginalised group of people to ensure that their experiences and perceptions of care are considered for service development. Over 100 people contributed their experiences of assessments and provided recommendations for improving practice. Participants were able to disclose their experiences in their own time, at their own discretion, and under their own control.

For peer review only - http://bmjopen.bmj.com/site/about/guidelines.xhtml

We have included a demographic profile of respondents to evaluate the relevance of our findings to clinical and carer groups. Our sample included a wide age range but only 17 participants were aged 18 to 25 and five were over 60. Experiences were similar across age groups, but future research is needed to explore issues for younger and older adults when accessing services. We did not include patients under the age of 18 because of distinctness of service provision for this demographic group. This is an important area for further research, given the transition between adolescent and adult services.[19] Consistent with previous studies of this topic, our sample was prominently comprised of White British females.[10-12] However, self-harm and suicide rates are rising amongst women.[20] Our results provide important information on the unmet needs for females attending the emergency department following self-harm. Our study included relatively small numbers of carers (14.8%), Black and minority ethnic groups (4%), and men (19.5%) which is a potential limitation of our recruitment approach. Whilst experiences of assessments were consistent across sociodemographic groups in our data, help-seeking and quality-of-care may vary considerably for some subgroups compared to White British females. Future studies specifically designed for these populations are important to ensure equitable access to services and tailored treatments.

This is a qualitative study based on patients' experiences and their perceptions of care that they received. We are unable to say if substandard or iatrogenically harmful care caused some participants to repeat self-harm. Randomised controlled trials and prospective observational studies are needed to understand the range of outcomes following assessments and test formal hypotheses. Perceptions of care may be affected by previous negative experiences, poor follow-up, and psychological distress. Some individuals may perceive staff negatively and disengage with the assessment process if they have previously experienced stigmatising attitudes and/or have low self-worth.[12] Participants may also be more likely to participate in a study if they have negative experiences of services. However, negative experiences provide important opportunities for learning and improving services. Participants also provided examples of what they find helpful and recommendations for improving

For peer review only - http://bmjopen.bmj.com/site/about/guidelines.xhtml

clinical practice; therefore, we can also learn from scenarios in which participants have reported positive experiences.

This is the largest qualitative study to have examined psychosocial assessment following self-harm presentations to the emergency department. Our study was strengthened by substantive patient and carer involvement throughout the research process. Analyses were conducted by a multidisciplinary team that included clinical and research expertise and people with lived experience. This enabled triangulation at a research and respondent level, which enhanced the robustness and validity of the findings.[21]

Our results provide rich and detailed information on the assessment process from a population at elevated risk of self-harm repetition and suicide.[1] We have addressed many limitations of previous studies in this area (e.g., small sample sizes and lack of demographic information)[10] and the lack of patient/carer involvement. Our broad recruitment strategy included hospitals and community groups to gather a wide range of experiences from around the United Kingdom.

Comparisons with existing research

There appears to be little change in patient experiences of emergency departments after self-harm over the last 15 years.[10-12] Consistent with Hunter et al.,[11] we found that assessments could positively or negatively affect hopelessness and engagement with services. Patients reported uncertainty about the purpose of the assessment and their experiences with aftercare were often negative. Similar to Owens et al.[22] our results highlight how the transition through the emergency department affects engagement, emotional states during and after the assessment, and future help-seeking. Participants also reported extensive experiences of stigmatising attitudes and poor communication during the assessment process. Similarly, our results indicate that negative interactions with staff contributed and reinforced feelings of shame and internalised stigma. The concerning incidents where people were not offered pain-relief for self-harm are consistent with what has been reported in other studies

For peer review only - http://bmjopen.bmj.com/site/about/guidelines.xhtml

previously.[10, 22] Future studies should focus on the relationship between such incidents and patient safety following self-harm. Similar to MacDonald and colleagues,[10] we found that transitions were apparently poorly managed for some participants, leading to uncertainty and feelings of abandonment by the end of the assessment.

Implications for clinical practice

The novel findings that have been uncovered by this large qualitative study of patient/carer experiences of psychosocial assessment in the emergency department following self-harm suggest that elements of current practice are contrary to the NICE clinical recommendations[2] and national quality standards (QS34).[23] This contrast suggests that more resources and training may be helpful in this area. Table 3 compares the study's findings and their potential implications for practice.

[insert table 3 around here]

Liaison psychiatry services have rapidly transformed to develop alternative models of care during the Covid-19 pandemic, including ward reconfigurations and diversions away from the emergency department.[8] Psychosocial assessments remain a core component of practice but the method and location of delivery may change (e.g., virtual/ telephone assessments). The Royal College of Psychiatrists recent survey of liaison psychiatry service changes during the covid-19 pandemic suggests that the stigmatisation of mental illness by acute hospital staff may increase during this time of change.[8] It is therefore imperative that we better understand and ameliorate the harmful impact of stigmatising attitudes, and that any service changes consider patient and carer experiences and perceptions of care.

For peer review only - http://bmjopen.bmj.com/site/about/guidelines.xhtml

Table 3 NICE quality standards for self-harm compared to the study results, and potential implications for practice

	NICE Quality standards [QS34]	Study results (N=102)	Potential implications for practice based on patient/carer recommendations
**1**	People who have harmed themselves are cared for with compassion and the same respect and dignity as any service user.	People experienced significant levels of stigmatising attitudes throughout the assessment process.	Given the levels of stigmatising attitudes reported by participants and elevated risk of suicide associated with self-harm and clinical populations (e.g., autism spectrum condition, obsessive compulsive disorder, post-traumatic stress disorder, personality disorders)[24] staff training may be helpful. Training may be more effective if tailored towards staff groups (e.g., acute staff, liaison psychiatry staff), co-designed and delivered by people with relevant lived experience.
**2**	People who have self-harmed have an initial assessment of physical health, mental state, safeguarding concerns, social circumstances, and risk of self-harm repetition or suicide.	Participants felt initial assessments were focused on their mental state without attention to their distress or reasons for self-harm.<br><br>Long waiting-times were compounded by the continued use of having to wait until the patient is 'medically cleared' prior to assessment	Emphatic care at initial stages may help to encourage people to stay for further assessment. Clear communication about the purpose of initial assessments, and roles of each staff member may clarify the process and expected outcomes.<br><br>Trained support workers or mental health volunteers could ease transitions by providing support, check-ins, and soothing aids where needed. Joint-working between acute and liaison staff from initial stages could help with patient flow, engagement, and experience.[25]
**3**	People who have self-harmed receive a comprehensive psychosocial assessment.	Participants were unsure of the purposes of the assessment. People hoped for help but often felt let down by the lack of therapeutic engagement and aftercare. The way the assessment was carried out affected how the person felt afterwards.	Clear communication about the process, purpose, and expectations for the assessment may help people to understand the process. Care and sensitivity could help people feel safer during this vulnerable stage. Collaborative assessments that focus on building a therapeutic relationship could engage people in the process and build up trust in mental health services.[26]

For peer review only - http://bmjopen.bmj.com/site/about/guidelines.xhtml

	NICE Quality standards [QS34]	Study results (N=102)	Potential implications for practice based on patient/carer recommendations
**4**	People who have self-harmed receive the monitoring that they need whilst in the healthcare setting, to reduce self-harm repetition risk.	Empathetic check-ins from staff or support workers helped to encourage people to stay for assessment and provide reassurance about the process. However, monitoring from security guards was experienced as perceived as coercive and affected engagement with the assessment process.	Our results suggest that the use of security guards to monitor patients may harmfully impact on levels of engagement and help-seeking. Other ways to check-in and monitor patients at risk may be helpful such as trained support workers and staff. Where detainment may be necessary; staff training in mental health, robust legal justification, and clear communication over the roles of the personnel involved may help to improve patient and staff experience.
**5**	People who have self-harmed are cared for in a safe physical environment while in the healthcare setting, in order to reduce self-harm repetition.	Participants described poor experiences of waiting for lengthy periods of time in unsafe healthcare environments.	Environments that are separate from the emergency department may be beneficial for physical and psychological safety. Having the opportunity to wait in separate environments or quite rooms could allow some recovery and distance from the noise and intensity of the emergency department, which may facilitate greater engagement.
**6**	People receive continuing support for self-harm have a discussion with their healthcare professional about the potential benefits of psychological interventions specifically structured for people who self-harm.	Follow-up care was a major source of disillusionment for participants. There was little discussion of psychological therapies. Long waiting times were common. Participants were desperate for help at the time of the assessment and for more therapeutic engagement.	Our results suggest that greater communication and transparency over psychological therapies and waiting times may be helpful. Therapeutic assessments and enhanced availability of psychological therapies delivered by the liaison psychiatry team may be beneficial for some patients and carers.
**7**	People receiving continuing support for self-harm and moving between mental health services have a collaboratively developed plan describing how support will be provided during the transition.	Poor transitions whilst in the emergency department and linking between services (e.g., primary-care, secondary-care) left many participants feeling distressed and abandoned. Some participants were unclear the role of care plans that had a directives for “do-not-assess.”	Our findings suggest that assessments should be offered for every hospital presenting self-harm episode and plans updated. Co-designed advance directives with accessible crisis may provide more control and understanding over assessment/treatment options for some patients.[26]<br><br>Given the challenges in accessing specialist services, emergency department presentations for self-harm provide an important

For peer review only - http://bmjopen.bmj.com/site/about/guidelines.xhtml

			opportunity for intervention at a time of crisis for the patient. Good quality, compassionate assessments, and collaboratively developed safety plans may help to ease acute distress and prevent repeat self-harm.[27] Training in clinician-patient communication may help to provide a shared understanding of patient issues, reduce miscommunication, and thereby enhance therapeutic engagement and quality-of-care. [28-29]

For peer review only

For peer review only - http://bmjopen.bmj.com/site/about/guidelines.xhtml

References

1. Carr M.J, Ashcroft DM, Kontopantelis E, While D, Awenat Y, Cooper J, et al. Premature death among primary care patients with a history of self-harm. Ann Fam Med. 2017a; 15(3): 246-254.
2. NICE. Self-harm. The NICE Guideline on Longer-term management. National Clinical Guideline Number 133. London: The British Psychological Society and The Royal College of Psychiatrists; 2011.
3. Olfson M, Wall M, Wang S, Crystal S, Gerhard T, & Blanco C. (2017). Suicide following deliberate self-harm. Am J Psychiatry. 2017; (8): 765-774.
4. Geulayov G, Casey D, McDonald KC, Foster P, Pritchard K, Wells C, et al. (2018). Incidence of suicide, hospital-presenting non-fatal self-harm, and community-occurring non-fatal self-harm in adolescents in England (the iceberg model of self-harm): a retrospective study. Lancet Psychiatry. 2018; 5(2): 167-174.
5. Department of Health (2019). Preventing Suicide in England: Fourth Progress Report of the Cross-Government Outcomes Strategy to Save Lives. London: HM Government.
6. Bergen H, Hawton K, Waters K, Cooper J, & Kapur N. Psychosocial assessment and repetition of self-harm: the significance of single and multiple repeat episode analyses. J Affect Disord 2010; 127(1): 257-265.
7. Steeg S, Emsley R, Carr M, Cooper J, & Kapur N. Routine hospital management of self-harm and risk of further self-harm: propensity score analysis using record-based cohort data. Psychol Med. 2018; 48(2): 315-326.
8. Royal College of Psychiatrists. Alternatives to emergency departments for mental health assessments during the COVID-19 pandemic. UK: Faculty of Liaison Psychiatry. 2020a
9. Holmes EA, O'Connor RC, Perry VH, Tracey I, Wessely S, Arseneault L et al. Multidisciplinary research priorities for the COVID-19 pandemic: a call for action for mental health science. Lancet Psychiatry. 2020; 7: 547-60.

For peer review only - http://bmjopen.bmj.com/site/about/guidelines.xhtml

10. MacDonald S, Sampson C, Turley R, Biddle L., Ring N, Begley R, et al. Patients' Experiences of Emergency Hospital Care Following Self-Harm: Systematic Review and Thematic Synthesis of Qualitative Research. Qual Health Res. 2020; 30(3): 471-485.

11. Hunter C, Chantler K, Kapur N, & Cooper J. Service user perspectives on psychosocial assessment following self-harm and its impact on further help-seeking: a qualitative study. J Affect Disord. 2013; 145(3): 315-323.

12. Horrocks J, Hughes J, Martin C, House A, & Owens D. Patient experiences of hospital care following self- harm—A qualitative study. UK: University of Leeds. 2005

13. Braun V, Clarke V, Boulton, E, Davey L, McEvoy C. (2020). The online survey as a qualitative research tool. Int J Soc Res Methodol [internet] 2020 cited 17 August 20] Available from: https://www-tandfonline-com.manchester.idm.oclc.org/doi/full/10.1080/13645579.2020.1805550

14. Kapur N, Gunnell D, Hawton K, Nadeem S, Khalil S, Longson D, et al. Messages from Manchester: pilot randomised controlled trial following self-harm. The BJ Psych. 2013; 203(1): 73-74.

15. Fereday J, & Muir-Cochrane E. Demonstrating rigor using thematic analysis: A hybrid approach of inductive and deductive coding and theme development. Int J Qual Methods. 2006: 5(1): 80–92. doi: 10.1177/160940690600500107

16. IBM Corp. Released 2013. IBM SPSS Statistics for Windows, Version 22.0. Armonk, NY: IBM Corp.

17. QSR International Pty Ltd. NVivo (Version 12), https://www.qsrinternational.com/nvivo-qualitative-data-analysis-software/home NVivo qualitative data analysis software; QSR International Pty Ltd. Version 11, 2015.

18. Pierce M, McManus S, Jessop C, John A, Hotopf M, Ford T, et al. Says who? The significance of sampling in mental health surveys during COVID-19. Lancet Psychiatry. 2020; 7, 7: 567-568.

19. Singh SP, Paul M, Ford T, Kramer T, Weaver T, McLaren, S, et al. Process, outcome and experience of transition from child to adult mental healthcare: multiperspective study. BJ Psych. 2010; 197(4): 305-312.

For peer review only - http://bmjopen.bmj.com/site/about/guidelines.xhtml

20. Department of Health and Social Care. The Women's Mental Health Taskforce. Final Report. UK: Department of Health. 2018.
21. Peters S. Qualitative Research Methods in Mental Health. Evid Based Ment Health. 2010; 13: 35-40.
22. Owens C, Hansford L, Sharkey S, & Ford T. "Needs and fears of young people presenting at accident and emergency department following an act of self-harm: secondary analysis of qualitative data." BJ Psych. 2016; 208: 286-291.
23. NICE. Self-harm Quality Standard (QS34). UK. National Institute for Health Research. 2013. https://www.nice.org.uk/guidance/qs34/resources/selfharm-pdf-2098606243525
24. Ropper A H, Fazel S, & Runeson B. Suicide. N Eng J Med. 2020; 38: 266-74.
25. Brown S, Eales S, Hayhurst C, Hood S, McAllister E, Potts S, & Thomson, A. Side by Side: A UK-wide consensus statement on working together to help patients with mental health needs in acute hospitals. UK: Royal College of Psychiatrists. 2020. https://www.rcpsych.ac.uk/docs/default-source/members/faculties/liaison-psychiatry/liaison-sidebyside.pdf
26. Palombini E, Richardson J, McAllister E, Veale D, & Thompson AB. (2020). When self-harm is about preventing harm: emergency department management of obsessive-compulsive disorder and associated harm. BJPsych Bull [internet]. 2020 [cited 2nd July]. Available from https://doi-org.manchester.idm.oclc.org/10.1192/bjb.2020.70.
27. Royal College of Psychiatrists. CR229: Self-harm and suicide in adults. Final report of the Patient Safety Group. UK: Royal College of Psychiatrists. 2020b. Available from: https://www.rcpsych.ac.uk/docs/default-source/improving-care/better-mh-policy/college-reports/cr229_self-harm-and-suicide.pdf
28. McCabe R, & Healey PG. Miscommunication in doctor–patient communication. Top Cogn Sci. 2018; 10(2), 409-424.

For peer review only - http://bmjopen.bmj.com/site/about/guidelines.xhtml

29. Parker D, Byng R, Dickens C, Kinsey D, & McCabe R. Barriers and facilitators to GP–patient communication about emotional concerns in UK primary care: a systematic review. Fam Pract. 2020: 1-11.

30.

For peer review only

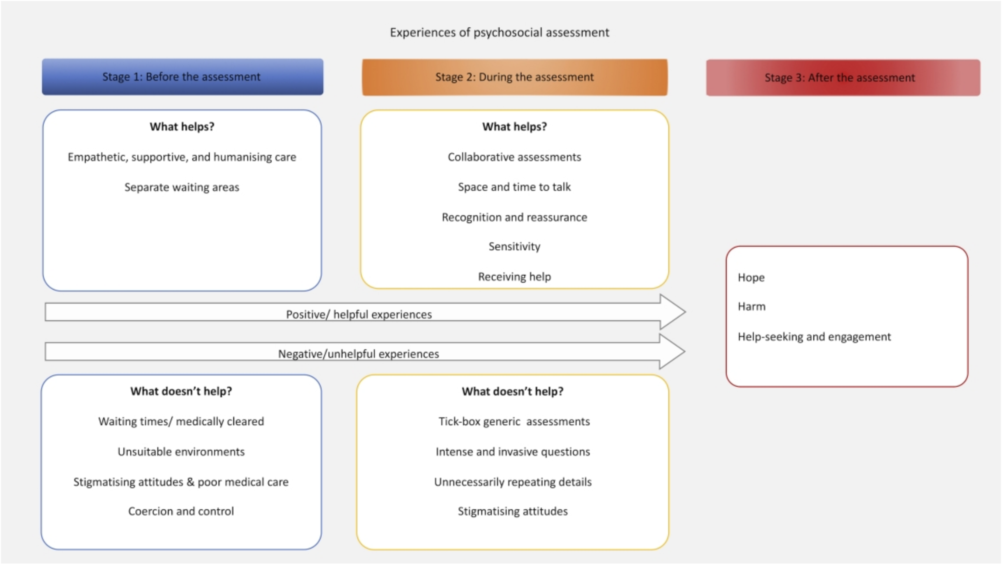

Figure 1. Themes and sub-themes across the three assessment process stages (1. Before the assessment; 2. During the assessment; 3. After the assessment)

338x190mm (300 x 300 DPI)

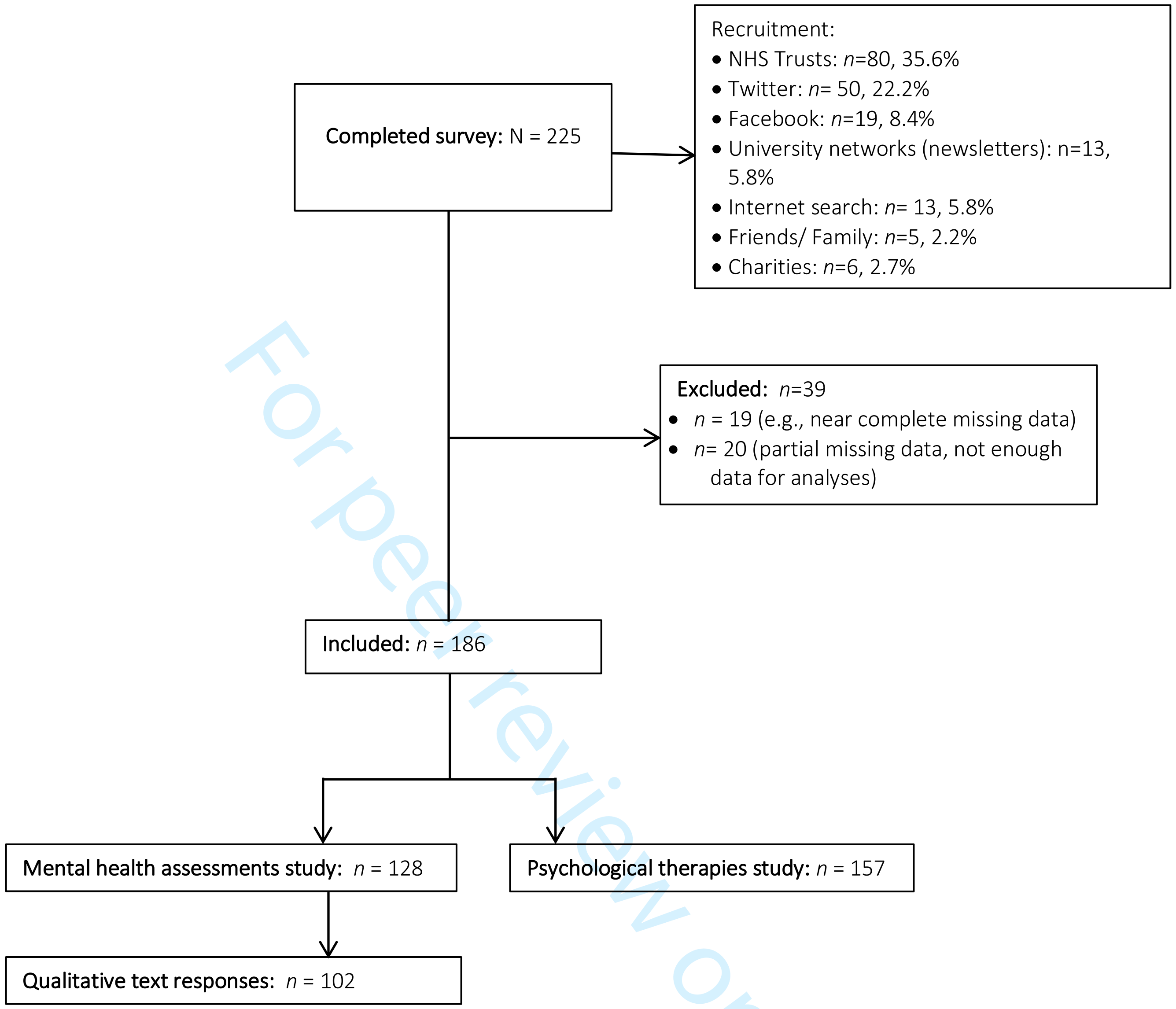

Figure 1

Flow chart of participants in the assessments and psychological therapies following self-harm. There were no relationships between missing data on age, sex, or ethnicity.

For peer review only

Appendix 2. Additional methodological details

Patient and public involvement

Our patient and carer advisory members were involved all aspects of the research process.[1] Two panel members (EM, SJB) with lived experience in this area contributed in-depth to the analyses and are co-authors of this paper. This research was also reviewed by a team with experience of mental health problems and their carers who have been specially trained to advise on research proposals and documentation through the Feasibility and Acceptability Support Team for Researchers (FAST-R): a free, confidential service in England provided by the National Institute for Health Research (NIHR) funded Maudsley Biomedical Research Centre via King's College London and South London and Maudsley NHS Foundation Trust. There was patient and public involvement input into our dissemination plan, which includes communicating key findings to relevant patient groups, carers, and mental health services.

Design and sample

Recruitment is often challenging for stigmatised topics and is particularly low for hospital presentations following self-harm.[2] An online survey, which was co-designed with patients and carers, enabled us to access a marginalised group of people who are often stigmatised, thereby providing greater anonymity and control to participants when sharing their experiences. [3]

Recruitment

This study was conducted as part of a larger programme of applied research investigating psychosocial assessments and psychological therapies following self-harm in the NIHR Greater Manchester Patient Safety Translational Research Centre. The survey was accessed through a University of Manchester website that contained a summary of the research process, a link to the participants' information sheet, consent form, and post-assessment debrief statement. We expected to recruit approximately

For peer review only

100 respondents, an estimate that was based on our previous studies that were conducted using online surveys.[4-5]

Online survey procedure

The study website, online survey tool, question format (e.g., yes/no, free-text; how the questions were asked), and questions were devised collaboratively with our patient/carer advisory panel via a series of workshops. We included open and closed text questions (e.g., demographics, selecting choices for services; binary response experiences) to elicit participant's views and experiences of assessments and follow-up care (see Appendix 2). We also asked for self-reported diagnosis, age, gender, education, and where the geographic area where the person resided. Survey questions for patients and carers were similar. Carers were invited to provide proxy information for diagnosis, living arrangements, and employment; and share their views of assessments for the person presenting with self-harm. Participants could tick boxes, skip questions, and/or complete free text responses without word limits. All participants provided informed consent for their participation. Due to the sensitive nature of the topic, links were provided to several online support sites after the assessment. Informed consent was obtained from all participants which was indicated by clicking continue on the survey.

For peer review only

Survey questions

- What is your experience of mental health assessments after you have presented to the emergency department with self-harm? Please tell us what worked well. Please tell us what worked less well.
- How did you feel about having the mental health assessment? How did you feel during the assessment? How did you feel after the assessment?
- Can you think of any reasons when you would be more likely to receive an assessment in the emergency department after self-harm?
- Can you think of any reasons when you would be less likely to receive an assessment following self-harm?
- Have you ever not received or been refused a mental health assessment following self-harm?
- Have you ever refused a mental health assessment?
- Have you ever left the emergency department before being assessed by a mental health clinician?
- Is there anything that would make it more likely that you would accept or stay for a mental health assessment?
- How would you change or do things differently?

Analysis: research team backgrounds

Data were analysed and interpreted by a multidisciplinary team that included expertise in psychiatry, mental health services research, psychology, and qualitative methodologies. The public contributors involved in the analyses included people with lived experience of self-harm and attending mental health services after harming themselves, as well as carers of these individuals.

w only

Participant demographics

The majority of patients were residing in England (99/102, 97.1%) and were of White British or Irish ethnicity (92/102, 91.1%). Most patients were either in paid or voluntary work (41/102, 41.4%), or were receiving statutory disability benefits (39/102, 39.4%); 9/102 (8.8%) were students and the remainder were not working (10/102, 10.1%). Many patients had a self-reported psychiatric diagnosis including the following disorders (that are not mutually exclusive): depression (60/102, 58.8%), emotionally unstable personality disorder (43/102, 42.2%), anxiety disorders (37, 36.3%), post-traumatic stress-disorder (25/102, 24.5%), eating disorders (13/102, 12.7%), psychotic disorders (10/102, 9.8%), bipolar disorder (10/102, 9.8%), autism spectrum disorder (7/102, 6.9%), obsessive compulsive disorder (6/102, 5.9%) dissociative disorders (4/102, 3.9%), and dysmorphic disorders (1/102, 1.0%).

Additional study limitations

We do not know how many participants had repeated attendances at an emergency department following a self-harm episode. Some participants reported that they had attended an emergency department several times following self-harm episodes and described positive and negative experiences. Most of the sample had a self-reported psychiatric illness diagnosis, and many had been diagnosed with a personality disorder or complex post-traumatic stress disorder. The needs and service experiences of these patients are important to capture given elevated suicide risk among people diagnosed with these conditions.[6] Further research is necessary to understand the needs of patients who present to the emergency department and do not have a psychiatric diagnosis or history of mental health service use. We did not include clinicians' perspectives in this study, but we are conducting another research to understand the barriers and facilitators to assessment and aftercare from their perspectives.

For peer review only

References

1. Littlewood, D. L., Quinlivan, L., Steeg, S., Bennett, C., Bickley, H., Rodway, C., ... & Kapur, N. (2019). Evaluating the impact of patient and carer involvement in suicide and self-harm research: A mixed-methods, longitudinal study protocol. *Health Expectations*, 00:1-7.
2. MacDonald, S., Sampson, C., Turley, R., Biddle, L., Ring, N., Begley, R., & Evans, R. (2020). Patients' Experiences of Emergency Hospital Care Following Self-Harm: Systematic Review and Thematic Synthesis of Qualitative Research. *Qualitative Health Research*, *30*(3), 471-485.
3. Braun, V., Clarke, V., Boulton, E., Davey, L., McEvoy, C. (2020). The online survey as a qualitative research tool. International Journal of Social Research Methodology, online 16/08/2020.
4. Quinlivan, L., Nowland, R., Steeg, S., Cooper, J., Meehan, D., Godfrey, J., ... & Allen, N. (2019). Advance decisions to refuse treatment and suicidal behaviour in emergency care: 'it's very much a step into the unknown'. *BJPsych open*, *5*(4).
5. Flynn, S., Raphael, J., Graney, J., Nyathi, T., Williams, A., Kapur, N., ... & Shaw, J. (2019). The personality disorder patient pathway: Service user and clinical perspectives. *Personality and mental health*, *13*(3), 134-143.
6. Doyle, M., While, D., Mok, P. L., Windfuhr, K., Ashcroft, D. M., Kontopantelis, E., ... & Webb, R. T. (2016). Suicide risk in primary care patients diagnosed with a personality disorder: a nested case control study. *BMC family practice*, *17*(1), 106.

Standards for Reporting Qualitative Research (SRQR)

O'Brien B.C., Harris, I.B., Beckman, T.J., Reed, D.A., & Cook, D.A. (2014). Standards for reporting qualitative research: a synthesis of recommendations. *Academic Medicine, 89(9)*, 1245-1251.

No. Topic	Item	Page number
**Title and abstract**		
S1 Title	Concise description of the nature and topic of the study identifying the study as qualitative or indicating the approach (e.g., ethnography, grounded theory) or data collection methods (e.g., interview, focus group) is recommended	Page 1
S2 Abstract	Summary of key elements of the study using the abstract format of the intended publication; typically includes objective, methods, results, and conclusions	Page 2
**Introduction**		
S3 Problem formulation	Description and significance of the problem/phenomenon studied; review of relevant theory and empirical work; problem statement	Page 4
S4 Purpose or research question	Purpose of the study and specific objectives or questions	Page 4-5
**Methods**		
S5 Qualitative approach and research paradigm	Qualitative approach (e.g., ethnography, grounded theory, case study, phenomenology, narrative research) and guiding theory if appropriate; identifying the research paradigm (e.g., positivist, constructivist/interpretivist) is also recommended	Page 6
S6 Researcher characteristics and reflexivity	Researchers' characteristics that may influence the research, including personal attributes, qualifications/experience, relationship with participants, assumptions, or presuppositions; potential or actual interaction between researchers' characteristics and the research	Appendix 2, page 3

For peer review only

	questions, approach, methods, results, or transferability	
S7 Context	Setting/site and salient contextual factors; rationale[a]	Page 6
S8 Sampling strategy	How and why research participants, documents, or events were selected; criteria for deciding when no further sampling was necessary (e.g., sampling saturation); rationale[a]	Page 6
S9 Ethical issues pertaining to human subjects	Documentation of approval by an appropriate ethics review board and participant consent, or explanation for lack thereof; other confidentiality and data security issues	Page 6 and in Appendix 2.
S10 Data collection methods	Types of data collected; details of data collection procedures including (as appropriate) start and stop dates of data collection and analysis, iterative process, triangulation of sources/methods, and modification of procedures in response to evolving study findings; rationale[a]	Page 6, 7 and in Appendix 2.
S11 Data collection instruments and technologies	Description of instruments (e.g., interview guides, questionnaires) and devices (e.g., audio recorders) used for data collection; if/how the instrument(s) changed over the course of the study	Page 6 and Appendix 2.
S12 Units of study	Number and relevant characteristics of participants, documents, or events included in the study; level of participation (could be reported in results)	Page 8. Additional information is presented in Appendix 2.
S13 Data processing	Methods for processing data prior to and during analysis, including transcription, data entry, data management and security, verification of data integrity, data coding, and anonymization/deidentification of excerpts	Page 7 and Appendix 2.
S14 Data analysis	Process by which inferences, themes, etc., were identified and developed, including researchers involved in data analysis; usually references a specific paradigm or approach; rationale[a]	Page 7 and Appendix 2.
S15 Techniques to enhance trustworthiness	Techniques to enhance trustworthiness and credibility of data analysis (e.g., member checking, audit trail, triangulation); rationale[a]	Page 4, page 7, Appendix 2, and the discussion on page 19.
**Results/Findings**		
S16 Synthesis and interpretation	Main findings (e.g., interpretations, inferences, and themes); might include development of a theory or model, or integration with prior research or theory	
S17 Links to empirical data	Evidence (e.g., quotes, field notes, text	

For peer review only

	excerpts, photographs) to substantiate analytic findings	
**Discussion**		
S18 Integration with prior work, implications, transferability, and contribution(s) to the field	Short summary of main findings; explanation of how findings and conclusions connect to, support, elaborate on, or challenge conclusions of earlier scholarship; discussion of scope of application/generalizability; identification of unique contribution(s) to scholarship in a discipline or field	Page 17
S19 Limitations	Trustworthiness and limitations of findings	Page 17
**Other**		
S20 Conflicts of interest	Potential sources of influence or perceived influence on study conduct and conclusions; how these were managed	In the statements section.
S21 Funding	Sources of funding and other support; role of funders in data collection, interpretation, and reporting	In the statements section.

[a]The rationale should briefly discuss the justification for choosing that theory, approach, method, or technique rather than other options available, the assumptions and limitations implicit in those choices, and how those choices influence study conclusions and transferability. As appropriate, the rationale for several items might be discussed together.

For peer review only

BMJ Open

"Relieved to be seen" - patient and carer experiences of mental health assessment in the emergency department following self-harm: qualitative analysis of 102 free-text survey responses

Journal:	*BMJ Open*
Manuscript ID	bmjopen-2020-044434.R1
Article Type:	Original research
Date Submitted by the Author:	28-Jan-2021

Complete List of Authors:	Quinlivan, Leah; The University of Manchester, Biology, Medicine, and Health<br>Gorman, Louise<br>Littlewood, Donna ; The University of Manchester,<br>Monaghan, Elizabeth<br>Barlow , Steven<br>Campbell, Stephen; The University of Manchester, Centre for Primary Care<br>Webb, Roger; The University of Manchester, School of Community Based Medicine<br>Kapur, Navneet; The University of Manchester, Centre for suicide prevention

**Primary Subject Heading**:	Health services research
Secondary Subject Heading:	Public health, Qualitative research, Mental health
Keywords:	Suicide & self-harm < PSYCHIATRY, Quality in health care < HEALTH SERVICES ADMINISTRATION & MANAGEMENT, PSYCHIATRY

SCHOLARONE™ Manuscripts

I, the Submitting Author has the right to grant and does grant on behalf of all authors of the Work (as defined in the below author licence), an exclusive licence and/or a non-exclusive licence for contributions from authors who are: i) UK Crown employees; ii) where BMJ has agreed a CC-BY licence shall apply, and/or iii) in accordance with the terms applicable for US Federal Government officers or employees acting as part of their official duties; on a worldwide, perpetual, irrevocable, royalty-free basis to BMJ Publishing Group Ltd ("BMJ") its licensees and where the relevant Journal is co-owned by BMJ to the co-owners of the Journal, to publish the Work in this journal and any other BMJ products and to exploit all rights, as set out in our licence.

The Submitting Author accepts and understands that any supply made under these terms is made by BMJ to the Submitting Author unless you are acting as an employee on behalf of your employer or a postgraduate student of an affiliated institution which is paying any applicable article publishing charge ("APC") for Open Access articles. Where the Submitting Author wishes to make the Work available on an Open Access basis (and intends to pay the relevant APC), the terms of reuse of such Open Access shall be governed by a Creative Commons licence – details of these licences and which Creative Commons licence will apply to this Work are set out in our licence referred to above.

Other than as permitted in any relevant BMJ Author's Self Archiving Policies, I confirm this Work has not been accepted for publication elsewhere, is not being considered for publication elsewhere and does not duplicate material already published. I confirm all authors consent to publication of this Work and authorise the granting of this licence.

For peer review only

Title: "Relieved to be seen" - patient and carer experiences of psychosocial assessment in the emergency department following self-harm: qualitative analysis of 102 free-text survey responses

Author details
Leah Quinlivan, PhD[1,2], Research Fellow; Louise Gorman, PhD[1], Research Associate; Donna Littlewood, PhD[1] Honorary Research Associate; Elizabeth Monaghan, Public contributor; Stephen J Barlow, Public Contributor; Stephen Campbell[3], Professor of Primary Care Research; Roger T. Webb, Professor of Mental Health Epidemiology;[1,2,3] Prof Nav Kapur, FRCPsych[1,2,3] Professor of Psychiatry and Population Health and Honorary Consultant Psychiatrist.

1. Centre for Mental Health and Safety, University of Manchester, England
2. Manchester Academic Health Science Centre, University of Manchester, England
3. NIHR Greater Manchester Patient Safety Translational Research Centre, The University of Manchester, Manchester academic Health Science Centre, Manchester, UK.
4. Greater Manchester Mental Health NHS Foundation Trust, Manchester, England

Corresponding author: Dr Leah Quinlivan
Address: Jean McFarlane Building, University of Manchester, Oxford Road, Manchester M13 9PL
Telephone: +44 (0)161 275 0727
Email: leah.quinlivan@manchester.ac.uk

For peer review only

ABSTRACT

Objectives: We sought to explore patient and carer experiences of psychosocial assessments following presentations to hospital after self-harm.

Design: Thematic analysis of free-text responses to an open-ended online survey.

Setting: Between March and November 2019, we recruited 88 patients (82% women) and 14 carers aged 18 or over from 16 English mental health trusts, community organisations, and via social media.

Results: Psychosocial assessments were experienced as helpful on some occasions but harmful on others. Participants' felt better, less suicidal, and less likely to repeat self-harm after good quality compassionate and supportive assessments. However, negative experiences during the assessment pathway were common and, in some cases, contributed to greater distress, less engagement, and further self-harm. Participants reported receiving negative and stigmatising comments about their injuries. Others reported that they were refused medical care or an anaesthetic. Stigmatising attitudes among some mental health staff centred on preconceived ideas over self-harm as a 'behavioural issue', inappropriate use of services, and psychiatric diagnosis.

Conclusion
Our findings highlight important patient experiences that can inform service provision and they demonstrate the value of involving patients/carers throughout the research process. Psychosocial assessments can be beneficial when empathetic and collaborative but less helpful when overly standardised, lacking in compassion, and waiting-times are unduly long. Patient views are essential to inform practice, particularly given the rapidly changing service context during and after the Covid-19 emergency.

Keywords: self-harm; suicide risk; liasion psychiatry; psychosocial assessments; qualitative research

For peer review only - http://bmjopen.bmj.com/site/about/guidelines.xhtml

Article summary

Strengths and limitations of this study

- Understanding what works and does not work for patients when receiving psychosocial assessments following self-harm is key to improving practice, however such evidence is limited.
- This is the largest qualitative study on psychosocial assessments following self-harm and the only study to have also included carer perspectives.
- Our extensive patient and carer involvement and use of a qualitative survey enabled us to access a marginalised and stigmatised group of patients and carers with substantive unmet healthcare needs.
- A limitation of this study is the use of a non-probability survey design. However, our aim was to provide qualitative experiential data and not to generalise to the broader population.
- Most of the respondents in the study were White British Women from England (72/88, 81.8%), who may differ from other patients who have limited literacy skills, or did not complete the survey in important ways such as help-seeking and healthcare needs.

For peer review only

Statements:

Funding statement
This work was funded by the National Institute for Health Research (NIHR) Greater Manchester Patient Safety Translational Research Centre. The views expressed are those of the authors(s) and not necessarily those of the NIHR or the Department of Health and Social Care.

Competing interests
NK is a member of the Department of Health's (England) National Suicide Prevention Advisory Group. He chaired the NICE guideline development group for the longer-term management of self-harm and the NICE Topic Expert Group (which developed the quality standards for self-harm services). NK is currently chair of the updated NICE guideline for Depression and Topic Advisor to the new NICE self-harm guideline. He is also supported by the Greater Manchester Mental Health NHS Foundation Trust.

Acknowledgements:
We wish to thank the participants who have taken the time to generously share their often-painful experiences, and also our patient and carer panel (Dawn Allen, Stephen Barlow, David Daniels, Dan Stears, Elizabeth Monaghan, Paula Mazzucco, Manoj Mistry, Tracy Neil, Fiona Naylor, Javed Rehamn, Jonathan Smith), for their involvement and insights throughout the process. We are also grateful to our GM NIHR PSTRC staff stakeholder panel and to the FAST-R panel for their input into the study. We also wish to thanks staff at the mental health trusts and clinical research networks, as well as community organisations for facilitating the research and assisting with the recruitment. Finally, we wish to thank the reviewers for enabling us to improve the manuscript with their helpful suggestions.

Authorship & contribution
All authors made substantial contributions to the study. SC, RW, and NK were responsible for funding acquisition. We designed and developed the study through discussion at team meetings with LQ, DL, RW, NK, and at meetings with members of our patient/ public involvement (PPI) group. LQ coordinated data collection with assistance from DL. LQ and LG did the analyses with input from our wider PPI panel, and from EM, SB, DL, RW, and NK. LQ interpreted the results with input from LG, EM, SB, DL, RW, NK, and LQ wrote the first draft. All authors contributed to subsequent drafts and approved the final version. All authors take responsibility for the integrity of the data and accuracy of the data analysis. NK is the guarantor of the study.

Data availability
The data that support the findings of this study are not publicly available due to restrictions of the research (consent and information that could compromise the privacy of some research participants).

Patient and public involvement
Our patient and carer advisory panel were involved all aspects of the research process including the design, conduct, reporting, and dissemination plans for the research. Two panel members (EM, SJB) with lived experience in this area contributed in-depth to the analyses and are co-authors of this paper. This research was also reviewed by a team with experience of mental health problems and their carers who have been specially trained to advise on research proposals and documentation through the Feasibility and Acceptability Support Team for Researchers (FAST-R): a free, confidential service in England provided by the National Institute for Health Research (NIHR) funded Maudsley Biomedical Research Centre via King's College London and South London and Maudsley NHS Foundation Trust. There was patient and public involvement input into our dissemination plan, which includes communicating key findings to relevant patient groups, carers, and mental health services.

For peer review only

INTRODUCTION

Self-harm is a common antecedent and strong risk factor for suicide.[1-3] Repeat self-harm occurs frequently and people who harm themselves more than once have an even higher risk of suicide.[3] Although hospital presentations represent the 'tip of the iceberg' for self-harm,[4] they provide an important opportunity for intervention via the provision of good quality care.[5] Liaison psychiatry services are an integral part of the self-harm care pathway.[2] Specialist teams are typically situated in acute hospitals and provide liaison care for patients on wards and in the emergency department.[2] Psychosocial assessments are a core component of care and are recommended for all patients presenting to hospital services having harmed themselves.[2] Good quality assessments may help to prevent repeat self-harm. [6-7]

Liaison psychiatry has rapidly transformed to manage the consequences of the covid-19 pandemic on service provision.[8] Mental health services are in flux and long-term changes are likely.[8] However, psychosocial assessments remain a core part of practice.[8] To ensure high quality care in liaison psychiatry, an enhanced understanding from the perspective of patients and carers as to what helps and does not help is now needed more than ever.[9]

Several studies of variable methodological quality and reporting standard have investigated healthcare service experiences following self-harm.[10] However, only two relatively small and geographically local studies have focussed on psychosocial assessments,[11-12] and none have included carer perspectives or involved patients and carers throughout the research process.

Between March and November 2019, we collaborated with mental health trusts in England and other community organisations to conduct a qualitative online patient and carer survey on psychosocial assessments in relation to self-harm. For the current study, the specific objective was to explore

For peer review only - http://bmjopen.bmj.com/site/about/guidelines.xhtml

positive and negative patient experiences of psychosocial assessments in the emergency department in a large sample of patients and carers.

METHODS

Ethics statement

The authors assert that all procedures contributing to this work comply with the ethical standards of the relevant national and institutional committees on human experimentation and with the Helsinki Declaration of 1975, as revised in 2008. All procedures involving human subjects/patients were approved by [Greater Manchester Central Research Ethics Committee (REC No: 18/NW/0839)].

Design and sample

Taking a pragmatist research approach,[13] we conducted a qualitative online survey[14] to investigate patient and carer experiences of assessments following self-harm. Pragmatism is a solution-focussed approach that prioritises the most appropriate methods to address research problems .[15] Our aim for this research was not to generalise to a wider population or to estimate prevalence, but to instead provide qualitative experiential data about the assessment process. Recruitment is often challenging for stigmatised groups of people and particularly so in relation to hospital presentations following self-harm.[10] An online survey, which was co-designed with a diverse panel of patients and carers, enabled us to access a marginalised group of people who are often stigmatised, thereby providing greater anonymity and control to participants when sharing their experiences.[14, 16]

Recruitment

We invited patients and carers aged 18 or over with experience of self-harm (defined as intentional self-poisoning or self-injury irrespective of suicidal intent[2]), psychosocial assessments, and psychological therapies to participate in a national online survey. We did not include people under the age of 18 because of differences in service provision for child and adolescent mental health services (CAMHS) in England.[17] To capture a wide range of experiences, we recruited participants through 16

For peer review only

NHS mental health trusts around England, social media (e.g., Twitter, Facebook), community organisations (e.g., charities, patient groups), and newsletters, from April 2019 through November 2019. We closed recruitment when there was a sufficient detailed, rich and range of responses for the free-text questions (determined by consensus between the research team and patient/carer advisory panel) and due to pragmatic constraints (e.g., study deadlines). Additional methodological information is presented in Appendix 1.

Analysis

We used thematic analysis to explore patterns across the dataset that represented participants' experiences.[14] Structured questions were used deductively to form the initial coding framework. Inductive methods were developed to capture additional codes and context across the data set.[14, 18] Our patient and carer advisory group coded the data and developed the coding framework, which was applied across the dataset. After immersion and familiarisation with the data, LG and LQ independently coded the full study dataset with this framework and reviewed convergence for codes and themes. Throughout the iterative coding process, the researchers reviewed and revised the newly developed inductive codes and the application of the coding framework. Working together with patients and carers, themes were constructed and revised from group discussion to enhance their clarity and relevance. We analysed responses from subgroups (e.g., patients/carers, gender, age-groups) together because the responses consistently overlapped. The final thematic structure and illustrative quotes were agreed through discussion amongst the team (LQ, LG, EM, DL, SB, RW, NK).

Descriptive quantitative analyses were performed using SPSS version 22;[19] NVivo 12 Software [20] was used for data management.

For peer review only

RESULTS

Descriptive quantitative results

In total, 102 participants provided text responses on experiences of psychosocial assessments in the online survey. The majority of participants were patients (88/102, 86.3%), and the remainder were carers (14/102, 13.7%). Patients were aged between 18 and 75 and their median age was 34. Carers were aged between and 73 and their median age was 56. Most patient (72/88, 81.8%) and carer (13/14, 92.9%) respondents were women.

Qualitative results

Experiences of psychosocial assessments

Psychosocial assessments are embedded in pathways of care when patients attend the emergency department after harming themselves. Participant experiences reflected the temporal and dynamic nature of presentations for self-harm (e.g., the assessment process may take place over several hours). In order to understand what helps and does not help for assessments, it was important that we considered the context of what went before and came after the assessment. To capture the context of psychosocial assessments, the themes are presented under stages of the care: (1) before the assessment; (2) during the assessment; and (3) after the assessment. Figure 1 presents the themes and subthemes throughout the process. Additional supporting quotations are in Tables 1, 2, and 3.

For peer review only

[INSERT FIGURE 1 HERE]

Figure 1. Themes and subthemes across the three assessment process stages (1. Before the assessment; 2. During the assessment; 3. After the assessment)

For peer review only

For peer review only - http://bmjopen.bmj.com/site/about/guidelines.xhtml

Themes

Stage 1. Before the psychosocial assessment: What helped?

Empathetic, supportive, and humanising care

Positive experiences of the pre-assessment process centred on empathetic, supportive emergency department staff who reassured participants and humanised the experience. Many people were anxious and uncertain about the waiting times and assessment process. Clear communication, check-ins from staff, and empathy helped to reassure and encourage people to stay for the assessment. Having support during the initial stages helped some participants feel "looked after by a member of staff" (R94, female, age 50, patient), which was helpful when they were distressed. Soothing aids such as heat packs and/or drinks and emotional support helped to humanise the process while participants waited for the assessment.

> "Other times I've been given a blanket and a hot drink, [the clinician] explained what will happen, and someone stays with me until the assessment happens" (R04, female, age 40, patient).

What did not help?

Waiting times/medically cleared

Most participants reported long and frustrating waiting times for their mental health assessment. There was little communication about time-frames, which left people unsure of what was happening, when they would be seen, and by whom, etc. Often it was not possible to speak to a mental health professional until the physical injury had been treated (medically cleared), which added further delays, distress, and frustration. Some participants were left alone for long periods of time whist they waited to see a mental health clinician leading to greater uncertainty about the process.

> "It was not possible to speak to someone until I was 'medically cleared'. This left a lot of time alone, without support even to ask the staff for a toothbrush or understand how long I'd be there" (R70, female, age 29, patient).

For peer review only - http://bmjopen.bmj.com/site/about/guidelines.xhtml

Emergency department environments

Some people were offered a separate waiting room and support whilst they waited for the assessment which was helpful. However, for others the emergency department environment felt physically and psychologically unsafe. The noise, intensity, and lack of privacy increased their distress, particularly where there was a lack of communication over the process and waiting times. Others waited in physically unsafe rooms for long periods of time. One patient reported harming herself with "sharp objects" left in a bin whilst she waited over five hours to see mental health five hours to see mental health practitioners (R01). One mother reported that her son harmed himself and required stitches in an unsafe "side room without scrutiny or supervision" (R100, female, age 73, carer).

> "You are placed in a very small, usually filthy room and are left for hours. Myself and my family waited in the room 8 hours once whilst they had to try and calm me down and prevent me from absconding" (R112, female, age 39, patient).

Stigmatising attitudes and poor medical care

Participants reported a lack of parity of esteem in the emergency department between self-harm and physical healthcare. Negative interactions with general medical staff while waiting for an assessment or during medical treatment for self-harm were reported as common. Some felt they had been treated with contempt and as a lower priority than those with physical health issues, especially during busy periods. Others reported receiving punitive medical care from some general medical staff which included insensitive and derogatory comments about their injuries. Some participants reported that they were refused pain-relief because they had harmed themselves: "You never had an anaesthetic when you cut so we won't give one now to suture your wounds!" (R08, female, age 75, patient). Others were refused medical treatment because it was felt that they would repeat self-harm: "I was told that it would be a waste of time because 'you'll just go and do it again, or cut through our stitches'" (R17, female, age 27, patient), leaving some people at risk of further physical health complications or infection.

For peer review only - http://bmjopen.bmj.com/site/about/guidelines.xhtml

Coercion and control

Some participants attended the emergency department because of family or the police. Coercive practice from staff whilst waiting for the assessment compounded their lack of control in the process. Some were threatened with police involvement or a criminal record if they did not comply with assessments, which served to heighten feelings of disempowerment: "I don't have a choice. They decide if I need it and need to stay or they'll call the police" (R105, female, age 31, patient). This was evident where security personnel of a different gender to the patient were present during toilet breaks, further exacerbating feelings of disempowerment. The use of coercion in the emergency department adversely affected some participants' engagement with the assessment process. One person described how they were more likely to refuse an assessment: "because of coercion and threat of police being called if I leave or being sectioned if I don't comply" (R06, female, age 29, patient). This disempowering approach at the initial stage of the assessment contributed to an avoidance of future help-seeking for some participants.

Table 1: Before the psychosocial assessment: Themes and exemplar quotations

**Psychosocial assessment stage**	Themes	Example quotes
**What helped?**	Empathetic, supportive, and humanising care	"I arrived at the emergency department at 2am after a breakdown and had to wait over 5 hours for the mental health team to see me on the day shift. I had self-harmed and told the staff this, and I told them that I was feeling suicidal. I didn't refer myself to the hospital and I wanted to leave during the 5-hour wait but I was encouraged to wait to see the doctor before I could leave. The nurses on shift were pleasant and one- on-one with me a few times, offered drinks, etc" (R86, female, age 19, patient).<br><br>"In the hospital they were amazing!! Very kind and helpful, looked after me extremely well and didn't make me feel like a burden at all" (R63, female, age 26, patient).
**What did not help?**	Waiting times/medically cleared	"If we had gone into A&E for self-harm, psychiatric liaison would refuse to see my daughter until she has completed her treatment, be it stitches, stapling or NAC. They would refuse to do an assessment until she was medically fit/cleared; we often had to wait 5, 6, 7 hours for someone to do a mental health assessment" (R20, female, age 58, carer).
	Emergency	"Had to share room with a man who was very intoxicated and who

For peer review only - http://bmjopen.bmj.com/site/about/guidelines.xhtml

	department environments	had soiled himself, which was very frightening as a 21-year-old female. Then was moved to corridor for some time, until being moved back to the waiting room once my obs were stable as no more space on the ward. I was not able to see the mental health team until 10am the following day and later found out they had gone home for the night" (R41, female, age 26, patient).
	Stigmatising attitudes and poor medical care	"in many cases, staff lacked compassion. Such as invalidating my distress, stigmatising responses such as 'wow you really meant to kill yourself, didn't you!!', exclaiming at the severity of my previous scarring and saying I was 'adding to the collection', saying that my pain threshold must be high and deciding not to give me any pain relief or medications when stitching or cleaning wounds (almost as if it was to be a punishment for self-harming), saying that I was 'wasting time' and other people had 'real' injuries"(R17, female, age 27, patient).
	Coercion and control	"Don't like it when male security escort me to toilet in case I abscond" (R01, female, age 60, patient).

Stage 2: During the assessment: What helped?

Collaborative assessments and engaged communication

The way the assessment was conducted made a major difference to the person's experience. Active listening, good communication, and engaging with the person facilitated the collaborative process during the clinical encounter. Assessments were more helpful when the person felt they were given control and choice (collaborative assessments). Participants felt heard and respected when they were included in decision making and understood what was happening in the assessment.

> "I got taken into a separate room to discuss my situation and the options available – the staff member listened well and took all my intentions seriously. I felt heard" (R113, female, age 36, patient).

Space and time to talk

Opportunity and time to talk about their distress was helpful for some participants. One participant reported that the assessment was the first time that he had spoken to anyone about his mental health or suicidal ideation (R04, male, age 42, patient). Other participants found having the space and time to talk helpful irrespective of the follow-up care that they received.

For peer review only - http://bmjopen.bmj.com/site/about/guidelines.xhtml

> "I knew I wouldn't get any help but at the same time it was good to have the opportunity to talk to someone" (R36, female, age 31, patient).

Compassion, recognition, and reassurance

Compassionate care was a key determinant of a helpful assessment. Many participants experienced internalised stigma, anxiety, and a range of intense emotions during the assessment. Some people felt shame and guilt about presenting to hospital with self-harm, which was exacerbated by poor interactions with some medical staff. Others were nervous about the consequences of the assessment and/or judgement from staff if they disclosed the intensity of their thoughts: "Uncomfortable. It's hard to open up and/or share thoughts others might find concerning/abnormal" (R69, male, age 29, patient).

Assessments worked well when mental healthcare staff recognised the distress that led to the participant's self-harm and their anxiety over the process. Assessments were helpful for many participants when they were reassured that they did the "right thing in coming to A&E" (R09, non-binary, age 18, patient). Kindness and consideration helped to establish a therapeutic relationship and alleviate some participants' anxiety during this sensitive time. Compassionate care and active listening from mental health staff helped to humanise the assessment experience and develop a therapeutic relationship.

> "It [the psychosocial assessment] was good if staff were calm, considerate, but most of all compassionate. If they took time to listen and understand" (R20, female, age 33, patient).

Help

Participants attended the emergency department to seek help after harming themselves. Assessments were evaluated more positively when participants received collaboratively developed safety plans, referrals to promptly accessible specialist services, or provided follow-up phone calls from mental health staff. Actionable outcomes from the assessment helped to legitimise and validate participants' distress and reasons for seeking help.

> "At last someone was trying to help" (R39, male, age 72, patient).

For peer review only - http://bmjopen.bmj.com/site/about/guidelines.xhtml

During the assessments: What did not help?

Generic tick-box assessments

Unhelpful assessments were overly standardised and generic for some participants. Some participants felt that the assessments were designed to fit their psychological distress and complex experiences into “neat little boxes” (R106, male, age 40, patient). In these instances, the focus was on ticking boxes rather than hearing the person had to say and little room was left to explore and assist the person with their immediate distress in this “bureaucratic and uncaring” process (R86, female, age 19, patient). The lack of opportunity to meaningfully talk about their distress negated the potential therapeutic value of the assessment for some people.

> “It was very superficial with little in-depth questioning. No real understanding that there was a real issue” (R61, female, age 50, carer)

Intense and invasive questions

Assessments could be intense and intrusive for some people. The combination of overly standardised assessments and a lack of therapeutic engagement made it difficult for some people to talk about their distress. In some cases, the intensity of the assessment left people feeling more vulnerable.

> “I've felt emotionally uncontained due to assessments being invasive and asking you to dredge up past unhappy and traumatic experiences. I've often left feeling worse than when I went in and it put me off going to the ED when I was in a crisis for over a year” (R47, female, age 25, patient).

Unnecessarily repeating details

For others, assessments placed too much emphasis on the past and did not focus on their current level of distress. Many shared their life experiences and the reasons behind their self-harm during previous assessments. Being asked to give this information again exacerbated the lack of continuity of care, assumptions that notes have not been read, and frustrations with the participants’ experience of continuously poor understanding of some mental health conditions among some staff.

> “Tedious having to explain 'whole' story each admission to a different hospital” (R28, female, 60, carer).

> “I felt like I had to go over my whole story in detail even though it could have been looked up on RIO. I found it quite intimidating” (R37, female, age 32).

For peer review only - http://bmjopen.bmj.com/site/about/guidelines.xhtml

Stigmatising attitudes during the assessment

Stigmatising attitudes changed slightly across the assessment stage. Misconceptions about self-harm and attention seeking were an issue throughout the process though. However, during the pre-assessment stage, stigmatising attitudes often focused on the act of self-harm and a lack of parity of esteem between self-harm and physical health injuries. Participants reported receiving negative comments about wasting healthcare resources and their injuries. During the assessment stage, stigmatising attitudes commonly focused on psychiatric diagnosis and patterns of service use for many people. Many felt judged during the assessment because of their self-harm, particularly if they had previously presented to services. Some participants described issues that centred around self-harm as a behaviour and that seeking help indicated "attention seeking behaviour" (e.g., R08, female, age 75, patient; R20, female, age 58, carer). Perceiving self-harm as a behaviour indicated that the person was at fault for hurting themselves with little regard for any trauma or other psychological determinants. Some participants felt that they were spoken to condescendingly because they were "misbehaving" (R95, female, age 29, patient).

Many participants with a diagnosis of personality disorder reported experiencing stigmatising attitudes from staff once they became aware of their diagnosis as if this indicated that they must be "attention seeking" and a "time-waster" when they sought help in the emergency department (R20, female, age 58; carer R17, Female, age 27, patient). Some participants reported that the compassion and interest in the person changed during the assessment because of their diagnosis. Many participants felt unsupported and judged because of their service use. Assessments were rushed, and their diagnosis negated any possible risk of further self-harm and suicide from the perspective of healthcare staff.

> "No real understanding that there was a real issue - felt like my daughter was being treated as an attention seeker" (R61, female, age 50, carer).

For peer review only - http://bmjopen.bmj.com/site/about/guidelines.xhtml

Table 2: During the psychosocial assessment: Themes and exemplar quotations		
	Themes	Example quotes
**During the assessment: What helped?**	Collaborative assessments and engaged communication	"In any situation, what works well is when I feel listened to and like I had some input and agreement into the decision and follow up and most importantly that I understood the situation and why it was happening" (R34, female, age 34, patient).
	Space and time to talk	"I was given a very quick psychiatric assessment in A&E. I was appreciative of being given some attention at the time as it was the first time I'd spoken about my mental health and self-harm/suicidal ideation. …Ideally it would be beneficial to be given some time/space to explore issues rather than feeling that they want you processed and out of the department as soon as possible (R04, male, age 42, patient).
	Recognition and reassurance	"The last two occasions I have had an assessment with a psychiatric-liaison practitioner, they have been really positive. I was made to feel as a human and felt as though how I was feeling was validated… Initially, I was nervous. I find talking openly very difficult and tend to fabricate answers due to being scared of the repercussions. The nurses I saw could sense my apprehensions but were encouraging with me to speak. Afterwards, I felt like I wanted them to be a constant part of my mental health recovery, knowing that the reason why I saw them was due to nearly losing my life. They temporarily restored my faith in the MH system" (R59, female, age 24, patient).
	Help	"I got taken into a separate room to discuss my situation and options available - the staff member listened well and took all my intentions seriously. I felt heard. I was sent home afterwards with a detailed "crisis plan of action" that I could read and share whenever things were getting difficult. This was helpful" (R113, female, age 36, patient).
**During the assessment: What did not help?**	Generic tick-box assessments	"What didn't work well was being told I would be okay, the nature of a checklist-like set of questions to evaluate someone's mental health left no room for me to really talk about how I was actually feeling" (R09, non-binary, age 18, patient).
	Intense and invasive questions	"These assessments were often quite intense and invasive, in so much as a professional with whom I would never again have contact sought to dredge up my entire life history, only to send me home feeling more unsettled than when I'd arrived…" (R101, female, age 31, patient).
	Unnecessarily repeating details	"Sometimes there is little point in repeating the reasons or trying to explain why I have self-harmed. People often don't understand it as it is linked to OCD. I get frustrated when people don't understand and it then makes it difficult to work with professionals" (R95, female, age 29, patient).
**During the assessment: What did not help?**	Stigmatising attitudes during the assessment	"What doesn't work is being told I am doing it for attention, and that they know better than me what is helpful, so they won't change the plan. The most unhelpful things are to be told that I didn't really want to kill myself because I'm not dead and that it is up to me if I kill myself." (R116, female, age 39, patient)<br><br>"When I have presented with a diagnosis of emotionally unstable personality disorder, triage was still quick, but staff have been cold and lacking in empathy and compassion. Assessments were treated, almost with boredom, and I've been discharged despite being a current risk of suicide or further self-harm." (R47, female, age 25, patient)

For peer review only - http://bmjopen.bmj.com/site/about/guidelines.xhtml

Stage 3: After the assessment: Consequences

Hope

Feelings after the assessment were closely related to the perceived quality of the assessment and support received during and afterwards. More positive emotions were reported after collaborative and compassionate assessments that provided practical help from the mental health clinician. Humanising care helped to lessen the person's distress and ease the acuity of their psychological crisis. Emotive challenges of the assessment process were offset for some because they received a first step towards seeking help and managing their self-harm. Humanising and supportive care helped to provide participants with hope.

> "After the assessments that were more caring and humanising, I tended better to my injuries, and felt less self-hatred. They were still deeply upsetting and difficult experiences, but I was more likely to be able to take steps toward self-care and getting help, and also less likely to self-harm in subsequent days" (R17, female, age 27, patient).

Harm

Most participants felt they would get help from the assessment but over a third indicated that they were harmed or felt worse by the process. Some reported feeling worse after the assessment because they experienced a lack of therapeutic engagement and support. Abrupt transitions out of the assessment left other people feeling abandoned and discarded. Positive effects of the assessment were negated for some people, with some participants reporting a worsening of their distress.

Negative and frustrating experiences of aftercare and support led many participants to perceive the assessments as a "waste of time" (R128, female, age 18, patient). Receiving out-of-date signposting information was frustrating for some people and others found the clinician's advice to be overly simplistic. Feeling unsupported during and after assessments led to despondency, hopelessness, and increased distress for some participants. In several instances, negative experiences of the assessment process and the lack of support led to further thoughts and acts of self-harm.

> "The assessment feels like, & is, a waste of time & makes you feel even more ashamed & worthless & more likely to self-harm again" (R21, female, age 47, patient).

For peer review only - http://bmjopen.bmj.com/site/about/guidelines.xhtml

Help-seeking and engagement

Experiences of attending the emergency department following self-harm had a cumulative effect and resulted in far-reaching consequences on help-seeking and engagement with mental health services for some participants. Some reported that the lack of a therapeutic assessment, inadequate follow-up care and/or long waiting lists to access support increased their levels of distress and disillusionment. Over a third of participants reported that they were less likely to seek help or engage with assessments and/or were more likely to leave the emergency department without an assessment.

> "I didn't feel like I was being treated as a very high priority, and went home feeling like there's no point in going to A&E when I'm suicidal from now on (even though that's what all health professionals seem to suggest)" (R15, male, age 22, patient).

Table 3: After the psychosocial assessment: Themes and exemplar quotations

**Themes**	
**Hope**	"before and during, I felt ashamed, like a fraud and saddened that I was putting my family and friends through all this, and even more so that I was now putting a drain on resources that could off been used for someone else who needed it more than I. After, I felt supported by family with me and the action plan we had all discussed. I felt there was a glimmer of hope in all the darkness" (R113, female, age 36, patient).
**Harm**	"I felt that there was no room to explore what was actually going on, that I was given little opportunity to express myself, and like the whole process was a bit of a tick-box exercise, rather than a supportive conversation or a way to come up with helpful suggestions. 9 times out of 10 these assessments left me feeling worse" (R101, female, age 31, patient).<br><br>"The RAID assessment made me feel listened to and gave me a next step. But the next step was pointless. I was back home feeling like nothing changed. So, I felt in many ways worse" (R70, female, age 29, patient).<br><br>"They tell you to simply go listen to music or have a bath and then discharge you and as a result, you attempt to end your life" (R30, female, age 24, patient).
**Help seeking and engagement**	" I felt judged whilst being asked questions about how I was feeling, and like I was being misunderstood and like I wasn't being taken seriously. And after the assessment and after discharge from A&E the vague advice I had been given made me even less likely to return to A&E in future" (R09, non-binary, age 18, patient).

For peer review only - http://bmjopen.bmj.com/site/about/guidelines.xhtml

DISCUSSION

Main findings

We found that the psychosocial assessment process was experienced as helpful on some occasions but harmful on others. Participants reported feeling better, less suicidal, and less likely to repeat self-harm after receiving compassionate care and collaborative assessments that focused on building a therapeutic relationship and providing help. Conversely, the pathway in the emergency department and the manner by which the assessment was conducted may have resulted in iatrogenic harm for some patients. Reports of substandard medical care and derogatory comments were common among participants, and compounded by what appear to be pre-conceived ideas among some staff members regarding service use patterns, self-harm repetition, and perceived attention-seeking behaviour. For some participants, cumulative experiences of long-waiting times, stigmatising attitudes, disjointed care, and overly standardised assessments contributed to heightened distress, disengagement, and in some cases further self-harm episodes.

Strengths and limitations

We conducted an online survey with non-probability sampling to recruit our participants, an approach that is subject to potential selection bias.[21] However, our aim was not to generalise to a broader population but to provide qualitative contextual experiences of attending the emergency department for psychosocial assessments. Self-harm is often stigmatised. Recruiting individuals who have harmed themselves can be challenging, especially for emergency department studies.[10-12,17] Our co-designed qualitative online survey enabled us to investigate a marginalised group of people to ensure that their experiences and perceptions of care are considered for service development. Over 100 people contributed their experiences of assessments and provided recommendations for improving practice. Participants were able to disclose their experiences in their own time, at their own discretion, and under their own control. Whilst the survey was designed with an emphasis on the generation of free text qualitative data, we were unable to probe responses or explore complex issues such as the

For peer review only - http://bmjopen.bmj.com/site/about/guidelines.xhtml

relationship between the socio-economic status of the patient and the quality of psychosocial assessments.

We have included a demographic profile of respondents to evaluate the relevance of our findings to clinical and carer groups. Our sample included a wide age range but only 17 (19.3%) of patients were aged 18 to 25 and five (5.7%) were over 60. Experiences were similar across age groups, but future research is needed to explore issues for younger and older adults when accessing services. We did not include patients under the age of 18 because of distinctness of service provision for this demographic group. This is an important area for further research, given the transition between adolescent and adult services.[22] Our sample is similar to Multicentre Monitoring Studies of Self-Harm in England[23] in terms of age, but we recruited more women, which is consistent with community surveys of self-harm.[10-12, 24] However, self-harm and suicide rates are rising amongst women.[25] Our results provide important information on the unmet needs for females attending the emergency department following self-harm. Our study included relatively small numbers of carers (13.7%), Black and minority ethnic groups (4%), and patients who were men (16%), which is similar to community studies of self-harm.[10, 24] Whilst experiences of assessments were consistent across sub-groups in our data, help-seeking and quality-of-care may vary considerably for some patients in these groups compared to White British females.

Only four participants had no self-reported psychiatric diagnosis (see Appendix 1) and many had been diagnosed with a personality disorder or complex post-traumatic stress disorder. Participants had all undergone psychosocial assessment, so may be more representative of patients with a greater level of need. Further research is needed to understand the needs of patients who present to hospital emergency departments and do not have a psychiatric diagnosis or history of mental health service use.

For peer review only - http://bmjopen.bmj.com/site/about/guidelines.xhtml

Qualitative surveys are a useful tool for gathering data on patient experiences.[14,16] However, there is potential to further exclude underrepresented populations in research, such as non-English speaking participants, or those with literacy issues, limited digital awareness or access, and people who do not wish to write about their experiences. Language around healthcare service use and help-seeking may also seem too abstract for some marginalised individuals. Further co-designed stand-alone studies and arts-led approaches specifically designed for these groups are indicated, to provide evidence for provision of more equitable access to services and better tailored treatments.

This is a qualitative study based on patients' experiences and their perceptions of care that they received. [sentences removed] Perceptions of care may be affected by previous negative experiences, poor follow-up, and psychological distress. Some individuals may perceive staff negatively and disengage with the assessment process if they have previously experienced stigmatising attitudes and/or have low self-worth.[12] Participants may also be more likely to participate in a study if they have negative experiences of services. However, negative experiences provide important opportunities for learning and improving services. Participants also provided examples of what they find helpful and recommendations for improving clinical practice; therefore, we can also learn from scenarios in which participants have reported positive experiences.

This is the largest qualitative study to have examined psychosocial assessment following self-harm presentations to the emergency department. Our study was strengthened by substantive patient and carer involvement throughout the research process. Analyses were conducted by a multidisciplinary team that included clinical and research expertise and people with lived experience. This enabled triangulation at a research and respondent level, which enhanced the robustness and validity of the findings.[26]

Our results provide rich and detailed information on the assessment process from a population at elevated risk of self-harm repetition and suicide.[1] We have addressed many limitations of previous

For peer review only

studies in this area (e.g., small sample sizes and lack of demographic information)[10] and the lack of patient/carer involvement. Our broad recruitment strategy included hospitals and community groups to gather a wide range of experiences from around the United Kingdom.

Comparisons with existing research

There appears to be little change in patient experiences of emergency departments after self-harm over the last 15 years.[10-12] Our findings are consistent with largely negative accounts of patients' experiences of 'hostile care' in emergency departments following self-harm.[27-34] For example, similar to our respondents, Pembroke[28] reported the harm arising from the lack of compassionate care in the emergency department:

> "The whole experience of Accident & Emergency is degrading for self-harmers. It just perpetuates the vicious circle. I am made to feel that I'm wasting their time and resources which results in me hating myself even more. This sustains my cutting. If only Accident & Emergency staff could realise that it would be easier for them and for us if they treated us humanely…" (Helen, p.23).[28]

The concerning incidents where people reported not being offered pain-relief for self-harm are consistent with what was found in other studies previously and patient reports. [10, 27, 32] Future studies should focus on the relationship between such incidents and patient safety following self-harm. Stigmatising attitudes of healthcare staff towards people who have harmed themselves and consequences for patients are well documented.[35-37] Similarly, our findings highlight pervasive negative attitudes towards people who have harmed themselves and also poor communication during the assessment process by some healthcare staff. They indicate that negative interactions with staff contributed and reinforced feelings of shame and internalised stigma. Further studies should explore the relationship between healthcare policies, cultures, systematic issues, and stigmatising attitudes towards people who have harmed themselves from a patient safety perspective.

Consistent with Hunter et al.,[11] we found that assessments could positively or negatively affect hopelessness and engagement with services. Patients reported uncertainty about the purpose of the assessment and their experiences with aftercare were often negative. Similar to Owens et al.[27] our

For peer review only - http://bmjopen.bmj.com/site/about/guidelines.xhtml

results highlight how the transition through the emergency department affects engagement, emotional states during and after the assessment, and future help-seeking. Similar to MacDonald and colleagues,[10] we found that transitions were apparently poorly managed for some participants, leading to uncertainty and feelings of abandonment by the end of the assessment.

Implications for clinical practice and policy

The novel findings that have been uncovered by this large qualitative study of patient/carer experiences of psychosocial assessment in the emergency department following self-harm suggest that elements of current practice are contrary to the NICE clinical recommendations[2] and national quality standards (QS34).[38] Greater public education on self-harm, clinical reflexivity, and good quality supervision may help to address some of the stigmatising attitudes in this area and improve patient experience. Table 3 compares the study's findings and their potential implications for practice.

Some of the poor experiences of care and assessment reported in this study possibly reflect a gap between current guidelines and their implementation, but others may reflect more fundamental challenges not just for guidelines but for clinical practice as a whole. For example, how do we balance the safety of service users with the provision of care that is as compassionate as it can be? What are the most appropriate outcome measures not just for self-harm research but for clinical services? The NICE guideline for the care of people who have harmed themselves are currently under revision and represent an opportunity to hopefully address some of these issues.

Measures defined by patients, other than risk of repeat self-harm and suicide may also help to improve patient experience.[39] To ensure cultural relevance, future co-designed studies should include the views of patients from different linguistic/ cultural backgrounds in policy developments (e.g., see Leckning and colleagues).[40] Further work is necessary to investigate clinician interpretation and implementation of guidelines in practice, alongside co-designed quality improvement initiatives which more closely evaluate patient experience in the emergency department.

For peer review only - http://bmjopen.bmj.com/site/about/guidelines.xhtml

Self-harm itself remains a policy priority, both as part of wider suicide prevention but also in its own right.[41] There needs to be a focus on research but also putting what we know into practice - assessments and evidence-based interventions should be available and accessible. Both staff training and service development must of course, be guided by those with lived experience.

[insert table 3 around here] .

Liaison psychiatry services have rapidly transformed to develop alternative models of care during the Covid-19 pandemic, including ward reconfigurations and diversions away from the emergency department.[8] Psychosocial assessments remain a core component of practice but the method and location of delivery may change (e.g., virtual/ telephone assessments). The Royal College of Psychiatrists recent survey of liaison psychiatry service changes during the covid-19 pandemic suggests that the stigmatisation of mental illness by acute hospital staff may increase during this time of change.[8] It is therefore imperative that we better understand and ameliorate the harmful impact of stigmatising attitudes, and that any service changes consider patient and carer experiences and perceptions of care.

For peer review only

Table 3 NICE quality standards for self-harm compared to the study results, and potential implications for practice

	NICE Quality standards [QS34]	Study results (N=102)	Potential implications for practice based on patient/carer recommendations
**1**	People who have harmed themselves are cared for with compassion and the same respect and dignity as any service user.	People experienced significant levels of stigmatising attitudes throughout the assessment process.	Given the levels of stigmatising attitudes reported by participants and elevated risk of suicide associated with self-harm and clinical populations (e.g., autism spectrum condition, obsessive compulsive disorder, post-traumatic stress disorder, personality disorders)[42] staff education that also considers reflexivity, culture, and socioeconomic factors may be helpful. Training may be more effective if tailored towards staff groups (e.g., acute staff, liaison psychiatry staff), co-designed and delivered by people with relevant lived experience.
**2**	People who have self-harmed have an initial assessment of physical health, mental state, safeguarding concerns, social circumstances, and risk of self-harm repetition or suicide.	Participants felt initial assessments were focused on their mental state without attention to their distress or reasons for self-harm.<br><br>Long waiting-times were compounded by the continued use of having to wait until the patient is 'medically cleared' prior to assessment	Emphatic care at initial stages may help to encourage people to stay for further assessment. Clear communication about the purpose of initial assessments, and roles of each staff member may clarify the process and expected outcomes.<br><br>Trained support workers or mental health volunteers could ease transitions by providing support, check-ins, and soothing aids where needed. Joint-working between acute and liaison staff from initial stages could help with patient flow, engagement, and experience.[43]
**3**	People who have self-harmed receive a comprehensive psychosocial assessment.	Participants were unsure of the purposes of the assessment. People hoped for help but often felt let down by the lack of therapeutic engagement and aftercare. The way the assessment was carried out affected how the person felt afterwards.	Clear communication about the process, purpose, and expectations for the assessment may help people to understand the process. Care and sensitivity could help people feel safer during this vulnerable stage. Collaborative assessments that focus on building a therapeutic relationship could engage people in the process and build up trust in mental health services.[44]

For peer review only - http://bmjopen.bmj.com/site/about/guidelines.xhtml

	NICE Quality standards [QS34]	Study results (N=102)	Potential implications for practice based on patient/carer recommendations
**4**	People who have self-harmed receive the monitoring that they need whilst in the healthcare setting, to reduce self-harm repetition risk.	Empathetic check-ins from staff or support workers helped to encourage people to stay for assessment and provide reassurance about the process. However, monitoring from security guards was experienced as perceived as coercive and affected engagement with the assessment process.	Our results suggest that the use of security guards to monitor patients may harmfully impact on levels of engagement and help-seeking. Other ways to check-in and monitor patients at risk may be helpful such as trained support workers and staff. Where detainment may be necessary; staff training in mental health, robust legal justification, and clear communication over the roles of the personnel involved may help to improve patient and staff experience.
**5**	People who have self-harmed are cared for in a safe physical environment while in the healthcare setting, in order to reduce self-harm repetition.	Participants described poor experiences of waiting for lengthy periods of time in unsafe healthcare environments.	Environments that are separate from the emergency department may be beneficial for physical and psychological safety. Having the opportunity to wait in separate environments or quite rooms could allow some recovery and distance from the noise and intensity of the emergency department, which may facilitate greater engagement.
**6**	People receive continuing support for self-harm have a discussion with their healthcare professional about the potential benefits of psychological interventions specifically structured for people who self-harm.	Follow-up care was a major source of disillusionment for participants. There was little discussion of psychological therapies. Long waiting times were common. Participants were desperate for help at the time of the assessment and for more therapeutic engagement.	Our results suggest that greater communication and transparency over psychological therapies and waiting times may be helpful. Therapeutic assessments and enhanced availability of psychological therapies delivered by the liaison psychiatry team may be beneficial for some patients and carers.
**7**	People receiving continuing support for self-harm and moving between mental health services have a collaboratively developed plan describing how support will be provided during the transition.	Poor transitions whilst in the emergency department and linking between services (e.g., primary-care, secondary-care) left many participants feeling distressed and abandoned. Some participants were unclear the role of care plans that had a directives for "do-not-assess."	Our findings suggest that assessments should be offered for every hospital presenting self-harm episode and plans updated. Co-designed advance directives with accessible crisis may provide more control and understanding over assessment/treatment options for some patients.[44]<br><br>Given the challenges in accessing specialist services, emergency department presentations for self-harm provide an important

For peer review only - http://bmjopen.bmj.com/site/about/guidelines.xhtml

			opportunity for intervention at a time of crisis for the patient. Good quality, compassionate assessments, and collaboratively developed safety plans may help to ease acute distress and prevent repeat self-harm.[45] Training in clinician-patient communication may help to provide a shared understanding of patient issues, reduce miscommunication, and thereby enhance therapeutic engagement and quality-of-care. [46-47]

For peer review only

References

1. Carr M.J, Ashcroft DM, Kontopantelis E, While D, Awenat Y, Cooper J, et al. Premature death among primary care patients with a history of self-harm. Ann Fam Med. 2017a; 15(3): 246-254.
2. NICE. Self-harm. The NICE Guideline on Longer-term management. National Clinical Guideline Number 133. London: The British Psychological Society and The Royal College of Psychiatrists; 2011.
3. Olfson M, Wall M, Wang S, Crystal S, Gerhard T, & Blanco C. (2017). Suicide following deliberate self-harm. Am J Psychiatry. 2017; (8): 765-774.
4. Geulayov G, Casey D, McDonald KC, Foster P, Pritchard K, Wells C, et al. (2018). Incidence of suicide, hospital-presenting non-fatal self-harm, and community-occurring non-fatal self-harm in adolescents in England (the iceberg model of self-harm): a retrospective study. Lancet Psychiatry. 2018; 5(2): 167-174.
5. Department of Health (2019). Preventing Suicide in England: Fourth Progress Report of the Cross-Government Outcomes Strategy to Save Lives. London: HM Government.
6. Bergen H, Hawton K, Waters K, Cooper J, & Kapur N. Psychosocial assessment and repetition of self-harm: the significance of single and multiple repeat episode analyses. J Affect Disord 2010; 127(1): 257-265.
7. Steeg S, Emsley R, Carr M, Cooper J, & Kapur N. Routine hospital management of self-harm and risk of further self-harm: propensity score analysis using record-based cohort data. Psychol Med. 2018; 48(2): 315-326.
8. Royal College of Psychiatrists. Alternatives to emergency departments for mental health assessments during the COVID-19 pandemic. UK: Faculty of Liaison Psychiatry. 2020a
9. Holmes EA, O'Connor RC, Perry VH, Tracey I, Wessely S, Arseneault L et al. Multidisciplinary research priorities for the COVID-19 pandemic: a call for action for mental health science. Lancet Psychiatry. 2020; 7: 547-60.

For peer review only - http://bmjopen.bmj.com/site/about/guidelines.xhtml

10. MacDonald S, Sampson C, Turley R, Biddle L., Ring N, Begley R, et al. Patients' Experiences of Emergency Hospital Care Following Self-Harm: Systematic Review and Thematic Synthesis of Qualitative Research. Qual Health Res. 2020; 30(3): 471-485.
11. Hunter C, Chantler K, Kapur N, & Cooper J. Service user perspectives on psychosocial assessment following self-harm and its impact on further help-seeking: a qualitative study. J Affect Disord. 2013; 145(3): 315-323.
12. Horrocks J, Hughes J, Martin C, House A, & Owens D. Patient experiences of hospital care following self- harm—A qualitative study. UK: University of Leeds. 2005.
13. Teddlie, C., & Tashakkori, (2015). Overview of contemporary issues in mixed methods research. In SAGE Handbook of Mixed Methods in Social and Behavioural Research. Thousand Oaks: SAGE Publications Inc.
14. Braun V, Clarke V, Boulton, E, Davey L, McEvoy C. (2020). The online survey as a qualitative research tool. Int J Soc Res Methodol [internet] 2020 cited 17 August 20] Available from: https://www-tandfonline-com.manchester.idm.oclc.org/doi/full/10.1080/13645579.2020.1805550
15. Kaushik V, Walsh CA. Pragmatism as a research paradigm and its implications for social work research. Social Sciences. 2019;8(9):255
16. Gibson, S., Boden, Z. V., Benson, O., & Brand, S. L. (2014). The impact of participating in suicide research online. *Suicide and Life-Threatening Behavior*, *44*(4), 372–383. doi:10.1111/sltb.12082
17. Kapur N, Gunnell D, Hawton K, Nadeem S, Khalil S, Longson D, et al. Messages from Manchester: pilot randomised controlled trial following self-harm. BJ Psych. 2013; 203(1): 73-74.
18. Fereday J, & Muir-Cochrane E. Demonstrating rigor using thematic analysis: A hybrid approach of inductive and deductive coding and theme development. Int J Qual Methods. 2006: 5(1): 80–92. doi: 10.1177/160940690600500107
19. IBM Corp. Released 2013. IBM SPSS Statistics for Windows, Version 22.0. Armonk, NY: IBM Corp.

For peer review only

20. QSR International Pty Ltd. NVivo (Version 12), https://www.qsrinternational.com/nvivo-qualitative-data-analysis-software/home NVivo qualitative data analysis software; QSR International Pty Ltd. Version 11, 2015.

21. Pierce M, McManus S, Jessop C, John A, Hotopf M, Ford T, et al. Says who? The significance of sampling in mental health surveys during COVID-19. Lancet Psychiatry. 2020; 7, 7: 567-568.

22. Singh SP, Paul M, Ford T, Kramer T, Weaver T, McLaren, S, et al. Process, outcome and experience of transition from child to adult mental healthcare: multiperspective study. BJ Psych. 2010; 197(4): 305-312.

23. Geulayov G, Kapur N, Turnbull P, Clements C, Waters K, Ness J, Townsend E, Hawton K. Epidemiology and trends in non-fatal self-harm in three centres in England, 2000–2012: findings from the Multicentre Study of Self-harm in England. BMJ Open. 2016 Apr 1;6(4).

24. Samaritans. Pushed from pillar to post. UK. Samaritans. 2020.

25. Department of Health and Social Care. The Women's Mental Health Taskforce. Final Report. UK: Department of Health. 2018.

26. Peters S. Qualitative Research Methods in Mental Health. Evid Based Ment Health. 2010; 13: 35-40.

27. Owens C, Hansford L, Sharkey S, & Ford T. "Needs and fears of young people presenting at accident and emergency department following an act of self-harm: secondary analysis of qualitative data". BJ Psych. 2016; 208: 286-291.

28. Pembroke. Self-harm: perspectives from personal experience, Survivors Speak Out. London. 1994.

29. Taylor TL, Hawton K, Fortune S, Kapur N. Attitudes towards clinical services among people who self-harm: systematic review. BJ Psych. 2009 Feb;194(2):104-10.

30. Chandler, A. Self-injury, medicine, and society: Authentic bodies. Palgrave Macmillan. 2016.

For peer review only - http://bmjopen.bmj.com/site/about/guidelines.xhtml

31. Hadfield J, Brown D, Pembroke L, Hayward M. Analysis of accident and emergency doctors' responses to treating people who self-harm. Qual Health Res. 2009;19(6):755-65.

32. DrEm_79. (2016). What your patient is thinking: Self-harm in the emergency department. 2016 *BMJ*, 353: 1150.

33. Chandler, A. (2018). Seeking secrecy: A qualitative study of younger adolescents' accounts of self-harm. *Young*, *26*(4), 313-331.

34. Harris J. Self-harm: Cutting the bad out of me. Qual Health Res. 2000;10(2):164-73.

35. Karman P, Kool N, Poslawsky IE, van Meijel B. Nurses' attitudes towards self-harm: a literature review. J Psychiatr Ment Health Nurs. 2015;22(1):65-75.

36. Rayner G, Blackburn J, Edward KL, Stephenson J, Ousey K. Emergency department nurse's attitudes towards patients who self-harm: A meta-analysis. Int J Ment Health Nurs. 2019;28(1):40-53.

37. Saunders KE, Hawton K, Fortune S, Farrell S. Attitudes and knowledge of clinical staff regarding people who self-harm: a systematic review. J Affect Disord Rep. 20121;139(3):205-16.

38. NICE. Self-harm Quality Standard (QS34). UK. National Institute for Health Research. 2013. https://www.nice.org.uk/guidance/qs34/resources/selfharm-pdf-2098606243525

39. Allen S. Self-harm and the words that bind: a critique of common perspectives. J Ment Heath Health Nurs. 2007;14(2):172-8.

40. Leckning B, Hirvonen T, Armstrong G, Carey TA, Westby M, Ringbauer A, Robinson G. Developing best practice guidelines for the psychosocial assessment of Aboriginal and Torres Strait Islander people presenting to hospital with self-harm and suicidal thoughts. Aust N Z J Psychiatry. 2020 Sep;54(9):874-82.

41. Kapur N. Services for self-harm: progress and promise? BJ Psych. 2020 Dec;217(6):663-4.

42. Ropper A H, Fazel S, & Runeson B. Suicide. N Eng J Med. 2020; 38: 266-74.

43. Brown S, Eales S, Hayhurst C, Hood S, McAllister E, Potts S, & Thomson, A. Side by Side: A UK-wide consensus statement on working together to help patients with mental health needs in

For peer review only - http://bmjopen.bmj.com/site/about/guidelines.xhtml

acute hospitals. UK: Royal College of Psychiatrists. 2020. https://www.rcpsych.ac.uk/docs/default-source/members/faculties/liaison-psychiatry/liaison-sidebyside.pdf

44. Palombini E, Richardson J, McAllister E, Veale D, & Thompson AB. (2020). When self-harm is about preventing harm: emergency department management of obsessive-compulsive disorder and associated harm. BJPsych Bull [internet]. 2020 [cited 2nd July]. Available from https://doi-org.manchester.idm.oclc.org/10.1192/bjb.2020.70.

45. Royal College of Psychiatrists. CR229: Self-harm and suicide in adults. Final report of the Patient Safety Group. UK: Royal College of Psychiatrists. 2020b. Available from: https://www.rcpsych.ac.uk/docs/default-source/improving-care/better-mh-policy/college-reports/cr229_self-harm-and-suicide.pdf

46. McCabe R, & Healey PG. Miscommunication in doctor–patient communication. Top Cogn Sci. 2018; 10(2), 409-424.

47. Parker D, Byng R, Dickens C, Kinsey D, & McCabe R. Barriers and facilitators to GP–patient communication about emotional concerns in UK primary care: a systematic review. Fam Pract. 2020: 1-11.

For peer review only - http://bmjopen.bmj.com/site/about/guidelines.xhtml

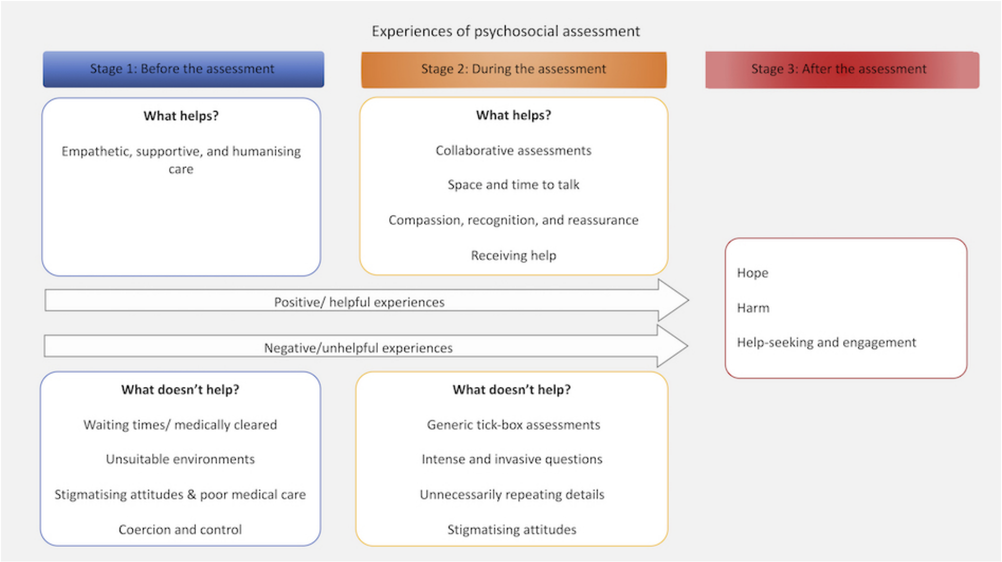

Figure 1. Themes and sub-themes across the three assessment process stages (1. Before the assessment; 2. During the assessment; 3. After the assessment)

80x44mm (300 x 300 DPI)

Appendix 1. Additional methodological details

Recruitment

This study was conducted as part of a larger programme of applied research investigating psychosocial assessments and psychological therapies following self-harm in the NIHR Greater Manchester Patient Safety Translational Research Centre. The survey was accessed through a University of Manchester website that contained a summary of the research process, a link to the participants' information sheet, consent form, and post-assessment debrief statement. We expected to recruit approximately 100 respondents, an estimate that was based on our previous studies that were conducted using online surveys.[2-3]

Online survey procedure

The study website, online survey tool, question format (e.g., yes/no, free-text; how the questions were asked), and questions were devised collaboratively with our patient/carer advisory panel via a series of workshops. We included open and closed text questions (e.g., demographics, selecting choices for services; binary response experiences) to elicit participant's views and experiences of assessments and follow-up care (see Box 1). We also asked for self-reported diagnosis, age, gender, education, and where the geographic area where the person resided. Survey questions for patients and carers were similar. Carers were invited to provide proxy information for diagnosis, living arrangements, and employment; and share their views of assessments for the person presenting with self-harm. Participants could tick boxes, skip questions, and/or complete free text responses without word limits. All participants provided informed consent for their participation. Due to the sensitive nature of the topic, links were provided to several online support sites after the assessment. Informed consent was obtained from all participants which was indicated by clicking continue on the survey.

For peer review only

Box 1. Survey questions

- What is your experience of mental health assessments after you have presented to the emergency department with self-harm? Please tell us what worked well. Please tell us what worked less well.
- How did you feel about having the mental health assessment? How did you feel during the assessment? How did you feel after the assessment?
- Can you think of any reasons when you would be more likely to receive an assessment in the emergency department after self-harm?
- Can you think of any reasons when you would be less likely to receive an assessment following self-harm?
- Have you ever not received or been refused a mental health assessment following self-harm?
- Have you ever refused a mental health assessment?
- Have you ever left the emergency department before being assessed by a mental health clinician?
- Is there anything that would make it more likely that you would accept or stay for a mental health assessment?
- How would you change or do things differently?

Data were analysed and interpreted by a multidisciplinary team that included expertise in psychiatry, mental health services research, psychology, and qualitative methodologies. The public contributors involved in the analyses included people with lived experience of self-harm and attending mental health services after harming themselves, as well as carers of these individuals.

Participant demographics

The majority of patients were residing in England (99/102, 97.1%) and were of White British or Irish ethnicity (92/102, 91.1%). Most patients were either in paid or voluntary work (41/102, 41.4%), or were receiving statutory disability benefits (39/102, 39.4%); 9/102 (8.8%) were students and the remainder were not working (10/102, 10.1%). Many patients had a self-reported psychiatric diagnosis

For peer review only - http://bmjopen.bmj.com/site/about/guidelines.xhtml

including the following disorders (that are not mutually exclusive): depression (60/102, 58.8%), emotionally unstable personality disorder (43/102, 42.2%), anxiety disorders (37/102, 36.3%), post-traumatic stress-disorder (25/102, 24.5%), eating disorders (13/102, 12.7%), psychotic disorders (10/102, 9.8%), bipolar disorder (10/102, 9.8%), autism spectrum disorder (7/102, 6.9%), obsessive compulsive disorder (6/102, 5.9%) dissociative disorders (4/102, 3.9%), and dysmorphic disorders (1/102, 1.0%).

References

1. Littlewood, D. L., Quinlivan, L., Steeg, S., Bennett, C., Bickley, H., Rodway, C., ... & Kapur, N. (2019). Evaluating the impact of patient and carer involvement in suicide and self-harm research: A mixed-methods, longitudinal study protocol. *Health Expectations*, 00:1-7.
2. Quinlivan, L., Nowland, R., Steeg, S., Cooper, J., Meehan, D., Godfrey, J., ... & Allen, N. (2019). Advance decisions to refuse treatment and suicidal behaviour in emergency care: ‘it's very much a step into the unknown’. *BJPsych open*, *5*(4).
3. Flynn, S., Raphael, J., Graney, J., Nyathi, T., Williams, A., Kapur, N., ... & Shaw, J. (2019). The personality disorder patient pathway: Service user and clinical perspectives. *Personality and mental health*, *13*(3), 134-143.

For peer review only

Standards for Reporting Qualitative Research (SRQR)

O'Brien B.C., Harris, I.B., Beckman, T.J., Reed, D.A., & Cook, D.A. (2014). Standards for reporting qualitative research: a synthesis of recommendations. *Academic Medicine, 89(9)*, 1245-1251.

No. Topic	Item	Page number
**Title and abstract**		
S1 Title	Concise description of the nature and topic of the study identifying the study as qualitative or indicating the approach (e.g., ethnography, grounded theory) or data collection methods (e.g., interview, focus group) is recommended	Page 1
S2 Abstract	Summary of key elements of the study using the abstract format of the intended publication; typically includes objective, methods, results, and conclusions	Page 2
**Introduction**		
S3 Problem formulation	Description and significance of the problem/phenomenon studied; review of relevant theory and empirical work; problem statement	Page 4
S4 Purpose or research question	Purpose of the study and specific objectives or questions	Page 4-5
**Methods**		
S5 Qualitative approach and research paradigm	Qualitative approach (e.g., ethnography, grounded theory, case study, phenomenology, narrative research) and guiding theory if appropriate; identifying the research paradigm (e.g., positivist, constructivist/interpretivist) is also recommended	Page 6
S6 Researcher characteristics and reflexivity	Researchers' characteristics that may influence the research, including personal attributes, qualifications/experience, relationship with participants, assumptions, or presuppositions; potential or actual interaction between researchers' characteristics and the research	Appendix 2, page 3

For peer review only

	questions, approach, methods, results, or transferability	
S7 Context	Setting/site and salient contextual factors; rationale[a]	Page 6
S8 Sampling strategy	How and why research participants, documents, or events were selected; criteria for deciding when no further sampling was necessary (e.g., sampling saturation); rationale[a]	Page 6
S9 Ethical issues pertaining to human subjects	Documentation of approval by an appropriate ethics review board and participant consent, or explanation for lack thereof; other confidentiality and data security issues	Page 6 and in Appendix 2.
S10 Data collection methods	Types of data collected; details of data collection procedures including (as appropriate) start and stop dates of data collection and analysis, iterative process, triangulation of sources/methods, and modification of procedures in response to evolving study findings; rationale[a]	Page 6, 7 and in Appendix 2.
S11 Data collection instruments and technologies	Description of instruments (e.g., interview guides, questionnaires) and devices (e.g., audio recorders) used for data collection; if/how the instrument(s) changed over the course of the study	Page 6 and Appendix 2.
S12 Units of study	Number and relevant characteristics of participants, documents, or events included in the study; level of participation (could be reported in results)	Page 8. Additional information is presented in Appendix 2.
S13 Data processing	Methods for processing data prior to and during analysis, including transcription, data entry, data management and security, verification of data integrity, data coding, and anonymization/deidentification of excerpts	Page 7 and Appendix 2.
S14 Data analysis	Process by which inferences, themes, etc., were identified and developed, including researchers involved in data analysis; usually references a specific paradigm or approach; rationale[a]	Page 7 and Appendix 2.
S15 Techniques to enhance trustworthiness	Techniques to enhance trustworthiness and credibility of data analysis (e.g., member checking, audit trail, triangulation); rationale[a]	Page 4, page 7, Appendix 2, and the discussion on page 19.
**Results/Findings**		
S16 Synthesis and interpretation	Main findings (e.g., interpretations, inferences, and themes); might include development of a theory or model, or integration with prior research or theory	
S17 Links to empirical data	Evidence (e.g., quotes, field notes, text	

For peer review only

	excerpts, photographs) to substantiate analytic findings	
**Discussion**		
S18 Integration with prior work, implications, transferability, and contribution(s) to the field	Short summary of main findings; explanation of how findings and conclusions connect to, support, elaborate on, or challenge conclusions of earlier scholarship; discussion of scope of application/generalizability; identification of unique contribution(s) to scholarship in a discipline or field	Page 17
S19 Limitations	Trustworthiness and limitations of findings	Page 17
**Other**		
S20 Conflicts of interest	Potential sources of influence or perceived influence on study conduct and conclusions; how these were managed	In the statements section.
S21 Funding	Sources of funding and other support; role of funders in data collection, interpretation, and reporting	In the statements section.

[a]The rationale should briefly discuss the justification for choosing that theory, approach, method, or technique rather than other options available, the assumptions and limitations implicit in those choices, and how those choices influence study conclusions and transferability. As appropriate, the rationale for several items might be discussed together.

For peer review only

BMJ Open

“Relieved to be seen” - patient and carer experiences of psychosocial assessment in the emergency department following self-harm: qualitative analysis of 102 free-text survey responses

Journal:	*BMJ Open*
Manuscript ID	bmjopen-2020-044434.R2
Article Type:	Original research
Date Submitted by the Author:	17-Feb-2021
Complete List of Authors:	Quinlivan, Leah; The University of Manchester, Biology, Medicine, and Health<br>Gorman, Louise<br>Littlewood, Donna ; The University of Manchester,<br>Monaghan, Elizabeth<br>Barlow , Steven<br>Campbell, Stephen; The University of Manchester, Centre for Primary Care<br>Webb, Roger; The University of Manchester, School of Community Based Medicine<br>Kapur, Navneet; The University of Manchester, Centre for suicide prevention
**Primary Subject Heading**:	Health services research
Secondary Subject Heading:	Public health, Qualitative research, Mental health
Keywords:	Suicide & self-harm < PSYCHIATRY, Quality in health care < HEALTH SERVICES ADMINISTRATION & MANAGEMENT, PSYCHIATRY

SCHOLARONE™
Manuscripts

I, the Submitting Author has the right to grant and does grant on behalf of all authors of the Work (as defined in the below author licence), an exclusive licence and/or a non-exclusive licence for contributions from authors who are: i) UK Crown employees; ii) where BMJ has agreed a CC-BY licence shall apply, and/or iii) in accordance with the terms applicable for US Federal Government officers or employees acting as part of their official duties; on a worldwide, perpetual, irrevocable, royalty-free basis to BMJ Publishing Group Ltd ("BMJ") its licensees and where the relevant Journal is co-owned by BMJ to the co-owners of the Journal, to publish the Work in this journal and any other BMJ products and to exploit all rights, as set out in our licence.

The Submitting Author accepts and understands that any supply made under these terms is made by BMJ to the Submitting Author unless you are acting as an employee on behalf of your employer or a postgraduate student of an affiliated institution which is paying any applicable article publishing charge ("APC") for Open Access articles. Where the Submitting Author wishes to make the Work available on an Open Access basis (and intends to pay the relevant APC), the terms of reuse of such Open Access shall be governed by a Creative Commons licence – details of these licences and which Creative Commons licence will apply to this Work are set out in our licence referred to above.

Other than as permitted in any relevant BMJ Author's Self Archiving Policies, I confirm this Work has not been accepted for publication elsewhere, is not being considered for publication elsewhere and does not duplicate material already published. I confirm all authors consent to publication of this Work and authorise the granting of this licence.

For peer review only

Title: "Relieved to be seen" - patient and carer experiences of psychosocial assessment in the emergency department following self-harm: qualitative analysis of 102 free-text survey responses

Author details
Leah Quinlivan, PhD[1,2], Research Fellow; Louise Gorman, PhD[1], Research Associate; Donna Littlewood, PhD[1] Honorary Research Associate; Elizabeth Monaghan, Public contributor; Stephen J Barlow, Public Contributor; Stephen Campbell[3], Professor of Primary Care Research; Roger T. Webb, Professor of Mental Health Epidemiology;[1,2,3] Prof Nav Kapur, FRCPsych[1,2,3] Professor of Psychiatry and Population Health and Honorary Consultant Psychiatrist.

1. Centre for Mental Health and Safety, University of Manchester, England
2. Manchester Academic Health Science Centre, University of Manchester, England
3. NIHR Greater Manchester Patient Safety Translational Research Centre, The University of Manchester, Manchester academic Health Science Centre, Manchester, UK.
4. Greater Manchester Mental Health NHS Foundation Trust, Manchester, England

Corresponding author: Dr Leah Quinlivan
Address: Jean McFarlane Building, University of Manchester, Oxford Road, Manchester M13 9PL
Telephone: +44 (0)161 275 0727
Email: leah.quinlivan@manchester.ac.uk

For peer review only

ABSTRACT

Objectives: We sought to explore patient and carer experiences of psychosocial assessments following presentations to hospital after self-harm.

Design: Thematic analysis of free-text responses to an open-ended online survey.

Setting: Between March and November 2019, we recruited 88 patients (82% women) and 14 carers aged 18 or over from 16 English mental health trusts, community organisations, and via social media.

Results: Psychosocial assessments were experienced as helpful on some occasions but harmful on others. Participants' felt better, less suicidal, and less likely to repeat self-harm after good quality compassionate and supportive assessments. However, negative experiences during the assessment pathway were common and, in some cases, contributed to greater distress, less engagement, and further self-harm. Participants reported receiving negative and stigmatising comments about their injuries. Others reported that they were refused medical care or an anaesthetic. Stigmatising attitudes among some mental health staff centred on preconceived ideas over self-harm as a 'behavioural issue', inappropriate use of services, and psychiatric diagnosis.

Conclusion
Our findings highlight important patient experiences that can inform service provision and they demonstrate the value of involving patients/carers throughout the research process. Psychosocial assessments can be beneficial when empathetic and collaborative but less helpful when overly standardised, lacking in compassion, and waiting-times are unduly long. Patient views are essential to inform practice, particularly given the rapidly changing service context during and after the Covid-19 emergency.

Keywords: self-harm; suicide risk; liasion psychiatry; psychosocial assessments; qualitative research

For peer review only

Article summary

Strengths and limitations of this study

- Understanding what works and does not work for patients when receiving psychosocial assessments following self-harm is key to improving practice, however such evidence is limited.
- This is the largest qualitative study on psychosocial assessments following self-harm and the only study to have also included carer perspectives.
- Our extensive patient and carer involvement and use of a qualitative survey enabled us to access a marginalised and stigmatised group of patients and carers with substantive unmet healthcare needs.
- A limitation of this study is the use of a non-probability survey design. However, our aim was to provide qualitative experiential data and not to generalise to the broader population.
- Most of the respondents in the study were White British Women from England (72/88, 81.8%), and their experiences may differ in important ways from other patients who have limited literacy skills, or did not complete the survey.

For peer review only

INTRODUCTION

Self-harm is a common antecedent and strong risk factor for suicide.[1-3] Repeat self-harm occurs frequently and people who harm themselves more than once have an even higher risk of suicide.[3] Although hospital presentations represent the 'tip of the iceberg' for self-harm,[4] they provide an important opportunity for intervention via the provision of good quality care.[5] Liaison psychiatry services are an integral part of the self-harm care pathway.[2] Specialist teams are typically situated in acute hospitals and provide liaison care for patients on wards and in the emergency department.[2] Psychosocial assessments are a core component of care and are recommended for all patients presenting to hospital services having harmed themselves.[2] Good quality assessments may help to prevent repeat self-harm. [6-7]

Liaison psychiatry has rapidly transformed to manage the consequences of the covid-19 pandemic on service provision.[8] Mental health services are in flux and long-term changes are likely.[8] However, psychosocial assessments remain a core part of practice.[8] To ensure high quality care in liaison psychiatry, an enhanced understanding from the perspective of patients and carers as to what helps and does not help is now needed more than ever.[9]

Several studies of variable methodological quality and reporting standard have investigated healthcare service experiences following self-harm.[10] However, only two relatively small and geographically local studies have focussed on psychosocial assessments,[11-12] and none have included carer perspectives or involved patients and carers throughout the research process.

Between March and November 2019, we collaborated with mental health trusts in England and other community organisations to conduct a qualitative online patient and carer survey on psychosocial assessments in relation to self-harm. For the current study, the specific objective was to explore

positive and negative patient experiences of psychosocial assessments in the emergency department in a large sample of patients and carers.

METHODS

Ethics statement

The authors assert that all procedures contributing to this work comply with the ethical standards of the relevant national and institutional committees on human experimentation and with the Helsinki Declaration of 1975, as revised in 2008. All procedures involving human subjects/patients were approved by [Greater Manchester Central Research Ethics Committee (REC No: 18/NW/0839)].

Design and sample

Taking a pragmatist research approach,[13] we conducted a qualitative online survey[14] to investigate patient and carer experiences of assessments following self-harm. Pragmatism is a solution-focussed approach that prioritises the most appropriate methods to address research problems .[15] Our aim for this research was not to generalise to a wider population or to estimate prevalence, but to instead provide qualitative experiential data about the assessment process. Recruitment is often challenging for stigmatised groups of people and particularly so in relation to hospital presentations following self-harm.[10] An online survey, which was co-designed with a diverse panel of patients and carers, enabled us to access a marginalised group of people who are often stigmatised, thereby providing greater anonymity and control to participants when sharing their experiences.[14, 16]

Recruitment

We invited patients and carers aged 18 or over with experience of self-harm (defined as intentional self-poisoning or self-injury irrespective of suicidal intent[2]), psychosocial assessments, and psychological therapies to participate in a national online survey. We did not include people under the age of 18 because of differences in service provision for child and adolescent mental health services (CAMHS) in England.[17] To capture a wide range of experiences, we recruited participants through 16

For peer review only - http://bmjopen.bmj.com/site/about/guidelines.xhtml

NHS mental health trusts around England, social media (e.g., Twitter, Facebook), community organisations (e.g., charities, patient groups), and newsletters, from April 2019 through November 2019. We closed recruitment when there was a sufficient detailed, rich and range of responses for the free-text questions (determined by consensus between the research team and patient/carer advisory panel) and due to pragmatic constraints (e.g., study deadlines). Additional methodological information is presented in Appendix 1.

Analysis

We used thematic analysis to explore patterns across the dataset that represented participants' experiences.[14] Structured questions were used deductively to form the initial coding framework. Inductive methods were developed to capture additional codes and context across the data set.[14, 18] Our patient and carer advisory group coded the data and developed the coding framework, which was applied across the dataset. After immersion and familiarisation with the data, LG and LQ independently coded the full study dataset with this framework and reviewed convergence for codes and themes. Throughout the iterative coding process, the researchers reviewed and revised the newly developed inductive codes and the application of the coding framework. Working together with patients and carers, themes were constructed and revised from group discussion to enhance their clarity and relevance. We analysed responses from subgroups (e.g., patients/carers, gender, age-groups) together because the responses consistently overlapped. The final thematic structure and illustrative quotes were agreed through discussion amongst the team (LQ, LG, EM, DL, SB, RW, NK).

Descriptive quantitative analyses were performed using SPSS version 22;[19] NVivo 12 Software [20] was used for data management.

Patient and public involvement

For peer review only

Our patient and carer advisory panel were involved all aspects of the research process including the design, conduct, reporting, and dissemination plans for the research. Two panel members (EM, SJB) with lived experience in this area contributed in-depth to the analyses and are co-authors of this paper. This research was also reviewed by a team with experience of mental health problems and their carers who have been specially trained to advise on research proposals and documentation through the Feasibility and Acceptability Support Team for Researchers (FAST-R): a free, confidential service in England provided by the National Institute for Health Research (NIHR) funded Maudsley Biomedical Research Centre via King's College London and South London and Maudsley NHS Foundation Trust. There was patient and public involvement input into our dissemination plan, which includes communicating key findings to relevant patient groups, carers, and mental health services.

RESULTS

Descriptive quantitative results

In total, 102 participants provided text responses on experiences of psychosocial assessments in the online survey. The majority of participants were patients (88/102, 86.3%), and the remainder were carers (14/102, 13.7%). Patients were aged between 18 and 75 and their median age was 34. Carers were aged between and 73 and their median age was 56. Most patient (72/88, 81.8%) and carer (13/14, 92.9%) respondents were women.

Qualitative results

<u>Experiences of psychosocial assessments</u>

Psychosocial assessments are embedded in pathways of care when patients attend the emergency department after harming themselves. Participant experiences reflected the temporal and dynamic nature of presentations for self-harm (e.g., the assessment process may take place over several hours). In order to understand what helps and does not help for assessments, it was important that

For peer review only - http://bmjopen.bmj.com/site/about/guidelines.xhtml

we considered the context of what went before and came after the assessment. To capture the context of psychosocial assessments, the themes are presented under stages of the care: (1) before the assessment; (2) during the assessment; and (3) after the assessment. Figure 1 presents the themes and subthemes throughout the process. Additional supporting quotations are in Tables 1, 2, and 3.

For peer review only

[INSERT FIGURE 1 HERE]

Figure 1. Themes and subthemes across the three assessment process stages (1. Before the assessment; 2. During the assessment; 3. After the assessment)

For peer review only

For peer review only - http://bmjopen.bmj.com/site/about/guidelines.xhtml

Themes

Stage 1. Before the psychosocial assessment: What helped?

Empathetic, supportive, and humanising care

Positive experiences of the pre-assessment process centred on empathetic, supportive emergency department staff who reassured participants and humanised the experience. Many people were anxious and uncertain about the waiting times and assessment process. Clear communication, check-ins from staff, and empathy helped to reassure and encourage people to stay for the assessment. Having support during the initial stages helped some participants feel "looked after by a member of staff" (R94, female, age 45-49, patient), which was helpful when they were distressed. Soothing aids such as heat packs and/or drinks and emotional support helped to humanise the process while participants waited for the assessment.

> "Other times I've been given a blanket and a hot drink, [the clinician] explained what will happen, and someone stays with me until the assessment happens" (R04, female, age 40-44, patient).

What did not help?

Waiting times/medically cleared

Most participants reported long and frustrating waiting times for their mental health assessment. There was little communication about time-frames, which left people unsure of what was happening, when they would be seen, and by whom, etc. Often it was not possible to speak to a mental health professional until the physical injury had been treated (medically cleared), which added further delays, distress, and frustration. Some participants were left alone for long periods of time whist they waited to see a mental health clinician leading to greater uncertainty about the process.

> "It was not possible to speak to someone until I was 'medically cleared'. This left a lot of time alone, without support even to ask the staff for a toothbrush or understand how long I'd be there" (R70, female, age 25-29, patient).

For peer review only - http://bmjopen.bmj.com/site/about/guidelines.xhtml

Emergency department environments

Some people were offered a separate waiting room and support whilst they waited for the assessment which was helpful. However, for others the emergency department environment felt physically and psychologically unsafe. The noise, intensity, and lack of privacy increased their distress, particularly where there was a lack of communication over the process and waiting times. Others waited in physically unsafe rooms for long periods of time. One patient reported harming herself with "sharp objects" left in a bin whilst she waited over five hours to see mental health five hours to see mental health practitioners (R01). One mother reported that her son harmed himself and required stitches in an unsafe "side room without scrutiny or supervision" (R100, female, age 70-74, carer).

> "You are placed in a very small, usually filthy room and are left for hours. Myself and my family waited in the room 8 hours once whilst they had to try and calm me down and prevent me from absconding" (R112, female, age 35-39, patient).

Stigmatising attitudes and poor medical care

Participants reported a lack of parity of esteem in the emergency department between self-harm and physical healthcare. Negative interactions with general medical staff while waiting for an assessment or during medical treatment for self-harm were reported as common. Some felt they had been treated with contempt and as a lower priority than those with physical health issues, especially during busy periods. Others reported receiving punitive medical care from some general medical staff which included insensitive and derogatory comments about their injuries. Some participants reported that they were refused pain-relief because they had harmed themselves: "You never had an anaesthetic when you cut so we won't give one now to suture your wounds!" (R08, female, age 70-74, patient). Others were refused medical treatment because it was felt that they would repeat self-harm: "I was told that it would be a waste of time because 'you'll just go and do it again, or cut through our stitches'" (R17, female, age 25-29, patient), leaving some people at risk of further physical health complications or infection.

For peer review only - http://bmjopen.bmj.com/site/about/guidelines.xhtml

Coercion and control

Some participants attended the emergency department because of family or the police. Coercive practice from staff whilst waiting for the assessment compounded their lack of control in the process. Some were threatened with police involvement or a criminal record if they did not comply with assessments, which served to heighten feelings of disempowerment: “I don’t have a choice. They decide if I need it and need to stay or they’ll call the police” (R105, female, age 30-34, patient). This was evident where security personnel of a different gender to the patient were present during toilet breaks, further exacerbating feelings of disempowerment. The use of coercion in the emergency department adversely affected some participants’ engagement with the assessment process. One person described how they were more likely to refuse an assessment: “because of coercion and threat of police being called if I leave or being sectioned if I don’t comply” (R06, female, age 25-29, patient). This disempowering approach at the initial stage of the assessment contributed to an avoidance of future help-seeking for some participants.

Table 1: Before the psychosocial assessment: Themes and exemplar quotations

Psychosocial assessment stage	Themes	Example quotes
**What helped?**	Empathetic, supportive, and humanising care	“I arrived at the emergency department at 2am after a breakdown and had to wait over 5 hours for the mental health team to see me on the day shift. I had self-harmed and told the staff this, and I told them that I was feeling suicidal. I didn’t refer myself to the hospital and I wanted to leave during the 5-hour wait but I was encouraged to wait to see the doctor before I could leave. The nurses on shift were pleasant and one- on-one with me a few times, offered drinks, etc” (R86, female, age 19-24, patient).<br><br>“In the hospital they were amazing!! Very kind and helpful, looked after me extremely well and didn't make me feel like a burden at all” (R63, female, age 25-29 patient).
**What did not help?**	Waiting times/medically cleared	“If we had gone into A&E for self-harm, psychiatric liaison would refuse to see my daughter until she has completed her treatment, be it stitches, stapling or NAC. They would refuse to do an assessment until she was medically fit/cleared; we often had to wait 5, 6, 7 hours for someone to do a mental health assessment” (R20, female, age 55-59, carer).
	Emergency	“Had to share room with a man who was very intoxicated and who

	department environments	had soiled himself, which was very frightening as a 21-year-old female. Then was moved to corridor for some time, until being moved back to the waiting room once my obs were stable as no more space on the ward. I was not able to see the mental health team until 10am the following day and later found out they had gone home for the night" (R41, female, age 25-29, patient).
	Stigmatising attitudes and poor medical care	"in many cases, staff lacked compassion. Such as invalidating my distress, stigmatising responses such as 'wow you really meant to kill yourself, didn't you!!', exclaiming at the severity of my previous scarring and saying I was 'adding to the collection', saying that my pain threshold must be high and deciding not to give me any pain relief or medications when stitching or cleaning wounds (almost as if it was to be a punishment for self-harming), saying that I was 'wasting time' and other people had 'real' injuries"(R17, female, age 25-29, patient).
	Coercion and control	"Don't like it when male security escort me to toilet in case I abscond" (R01, female, age 60-65, patient).

Stage 2: During the assessment: What helped?

Collaborative assessments and engaged communication

The way the assessment was conducted made a major difference to the person's experience. Active listening, good communication, and engaging with the person facilitated the collaborative process during the clinical encounter. Assessments were more helpful when the person felt they were given control and choice (collaborative assessments). Participants felt heard and respected when they were included in decision making and understood what was happening in the assessment.

> "I got taken into a separate room to discuss my situation and the options available – the staff member listened well and took all my intentions seriously. I felt heard" (R113, female, age 35-39, patient).

Space and time to talk

Opportunity and time to talk about their distress was helpful for some participants. One participant reported that the assessment was the first time that he had spoken to anyone about his mental health or suicidal ideation (R04, male, age 40-44, patient). Other participants found having the space and time to talk helpful irrespective of the follow-up care that they received.

For peer review only - http://bmjopen.bmj.com/site/about/guidelines.xhtml

> "I knew I wouldn't get any help but at the same time it was good to have the opportunity to talk to someone" (R36, female, age 30-34, patient).

Compassion, recognition, and reassurance

Compassionate care was a key determinant of a helpful assessment. Many participants experienced internalised stigma, anxiety, and a range of intense emotions during the assessment. Some people felt shame and guilt about presenting to hospital with self-harm, which was exacerbated by poor interactions with some medical staff. Others were nervous about the consequences of the assessment and/or judgement from staff if they disclosed the intensity of their thoughts: "Uncomfortable. It's hard to open up and/or share thoughts others might find concerning/abnormal" (R69, male, age 25-29, patient).

Assessments worked well when mental healthcare staff recognised the distress that led to the participant's self-harm and their anxiety over the process. Assessments were helpful for many participants when they were reassured that they did the "right thing in coming to A&E" (R09, non-binary, age 18-24, patient). Kindness and consideration helped to establish a therapeutic relationship and alleviate some participants' anxiety during this sensitive time. Compassionate care and active listening from mental health staff helped to humanise the assessment experience and develop a therapeutic relationship.

> "It [the psychosocial assessment] was good if staff were calm, considerate, but most of all compassionate. If they took time to listen and understand" (R20, female, age 30-34, patient).

Help

Participants attended the emergency department to seek help after harming themselves. Assessments were evaluated more positively when participants received collaboratively developed safety plans, referrals to promptly accessible specialist services, or provided follow-up phone calls from mental health staff. Actionable outcomes from the assessment helped to legitimise and validate participants' distress and reasons for seeking help.

> "At last someone was trying to help" (R39, male, age 70-74, patient).

For peer review only - http://bmjopen.bmj.com/site/about/guidelines.xhtml

During the assessments: What did not help?

Generic tick-box assessments

Unhelpful assessments were overly standardised and generic for some participants. Some participants felt that the assessments were designed to fit their psychological distress and complex experiences into "neat little boxes" (R106, male, age 40-44, patient). In these instances, the focus was on ticking boxes rather than hearing the person had to say and little room was left to explore and assist the person with their immediate distress in this "bureaucratic and uncaring" process (R86, female, age 18-24, patient). The lack of opportunity to meaningfully talk about their distress negated the potential therapeutic value of the assessment for some people.

> "It was very superficial with little in-depth questioning. No real understanding that there was a real issue" (R61, female, age 50-54, carer)

Intense and invasive questions

Assessments could be intense and intrusive for some people. The combination of overly standardised assessments and a lack of therapeutic engagement made it difficult for some people to talk about their distress. In some cases, the intensity of the assessment left people feeling more vulnerable.

> "I've felt emotionally uncontained due to assessments being invasive and asking you to dredge up past unhappy and traumatic experiences. I've often left feeling worse than when I went in and it put me off going to the ED when I was in a crisis for over a year" (R47, female, age 25-29, patient).

Unnecessarily repeating details

For others, assessments placed too much emphasis on the past and did not focus on their current level of distress. Many shared their life experiences and the reasons behind their self-harm during previous assessments. Being asked to give this information again exacerbated the lack of continuity of care, assumptions that notes have not been read, and frustrations with the participants' experience of continuously poor understanding of some mental health conditions among some staff.

> "Tedious having to explain 'whole' story each admission to a different hospital" (R28, female, age 60-64, carer).

For peer review only - http://bmjopen.bmj.com/site/about/guidelines.xhtml

> "I felt like I had to go over my whole story in detail even though it could have been looked up on RIO. I found it quite intimidating" (R37, female, age 30-34).

Stigmatising attitudes during the assessment

Stigmatising attitudes changed slightly across the assessment stage. Misconceptions about self-harm and attention seeking were an issue throughout the process though. However, during the pre-assessment stage, stigmatising attitudes often focused on the act of self-harm and a lack of parity of esteem between self-harm and physical health injuries. Participants reported receiving negative comments about wasting healthcare resources and their injuries. During the assessment stage, stigmatising attitudes commonly focused on psychiatric diagnosis and patterns of service use for many people. Many felt judged during the assessment because of their self-harm, particularly if they had previously presented to services. Some participants described issues that centred around self-harm as a behaviour and that seeking help indicated "attention seeking behaviour" (e.g., R08, female, age 75-79, patient; R20, female, age 55-59, carer). Perceiving self-harm as a behaviour indicated that the person was at fault for hurting themselves with little regard for any trauma or other psychological determinants. Some participants felt that they were spoken to condescendingly because they were "misbehaving" (R95, female, age 25-29, patient).

Many participants with a diagnosis of personality disorder reported experiencing stigmatising attitudes from staff once they became aware of their diagnosis as if this indicated that they must be "attention seeking" and a "time-waster" when they sought help in the emergency department (R20, female, age 55-59; carer R17, Female, age 25-29, patient). Some participants reported that the compassion and interest in the person changed during the assessment because of their diagnosis. Many participants felt unsupported and judged because of their service use. Assessments were rushed, and their diagnosis negated any possible risk of further self-harm and suicide from the perspective of healthcare staff.

> "No real understanding that there was a real issue - felt like my daughter was being treated as an attention seeker" (R61, female, age 50-54, carer).

For peer review only - http://bmjopen.bmj.com/site/about/guidelines.xhtml

Table 2: During the psychosocial assessment: Themes and exemplar quotations

	Themes	Example quotes
**During the assessment: What helped?**	Collaborative assessments and engaged communication	"In any situation, what works well is when I feel listened to and like I had some input and agreement into the decision and follow up and most importantly that I understood the situation and why it was happening" (R34, female, age 30-34, patient).
	Space and time to talk	"I was given a very quick psychiatric assessment in A&E. I was appreciative of being given some attention at the time as it was the first time I'd spoken about my mental health and self-harm/suicidal ideation. …Ideally it would be beneficial to be given some time/space to explore issues rather than feeling that they want you processed and out of the department as soon as possible (R04, male, age 40-44, patient).
	Recognition and reassurance	"The last two occasions I have had an assessment with a psychiatric-liaison practitioner, they have been really positive. I was made to feel as a human and felt as though how I was feeling was validated… Initially, I was nervous. I find talking openly very difficult and tend to fabricate answers due to being scared of the repercussions. The nurses I saw could sense my apprehensions but were encouraging with me to speak. Afterwards, I felt like I wanted them to be a constant part of my mental health recovery, knowing that the reason why I saw them was due to nearly losing my life. They temporarily restored my faith in the MH system" (R59, female, age 20-24, patient).
	Help	"I got taken into a separate room to discuss my situation and options available - the staff member listened well and took all my intentions seriously. I felt heard. I was sent home afterwards with a detailed "crisis plan of action" that I could read and share whenever things were getting difficult. This was helpful" (R113, female, age 35-39, patient).
**During the assessment: What did not help?**	Generic tick-box assessments	"What didn't work well was being told I would be okay, the nature of a checklist-like set of questions to evaluate someone's mental health left no room for me to really talk about how I was actually feeling" (R09, non-binary, age 18-24, patient).
	Intense and invasive questions	"These assessments were often quite intense and invasive, in so much as a professional with whom I would never again have contact sought to dredge up my entire life history, only to send me home feeling more unsettled than when I'd arrived…" (R101, female, age 30-34, patient).
	Unnecessarily repeating details	"Sometimes there is little point in repeating the reasons or trying to explain why I have self-harmed. People often don't understand it as it is linked to OCD. I get frustrated when people don't understand and it then makes it difficult to work with professionals" (R95, female, age 25-29, patient).
**During the assessment: What did not help?**	Stigmatising attitudes during the assessment	"What doesn't work is being told I am doing it for attention, and that they know better than me what is helpful, so they won't change the plan. The most unhelpful things are to be told that I didn't really want to kill myself because I'm not dead and that it is up to me if I kill myself." (R116, female, age 35-39, patient)<br><br>"When I have presented with a diagnosis of emotionally unstable personality disorder, triage was still quick, but staff have been cold and lacking in empathy and compassion. Assessments were treated, almost with boredom, and I've been discharged despite being a current risk of suicide or further self-harm." (R47, female, age 25-29, patient)

For peer review only - http://bmjopen.bmj.com/site/about/guidelines.xhtml

Stage 3: After the assessment: Consequences

Hope

Feelings after the assessment were closely related to the perceived quality of the assessment and support received during and afterwards. More positive emotions were reported after collaborative and compassionate assessments that provided practical help from the mental health clinician. Humanising care helped to lessen the person's distress and ease the acuity of their psychological crisis. Emotive challenges of the assessment process were offset for some because they received a first step towards seeking help and managing their self-harm. Humanising and supportive care helped to provide participants with hope.

> "After the assessments that were more caring and humanising, I tended better to my injuries, and felt less self-hatred. They were still deeply upsetting and difficult experiences, but I was more likely to be able to take steps toward self-care and getting help, and also less likely to self-harm in subsequent days" (R17, female, age 25-29, patient).

Harm

Most participants felt they would get help from the assessment but over a third indicated that they were harmed or felt worse by the process. Some reported feeling worse after the assessment because they experienced a lack of therapeutic engagement and support. Abrupt transitions out of the assessment left other people feeling abandoned and discarded. Positive effects of the assessment were negated for some people, with some participants reporting a worsening of their distress.

Negative and frustrating experiences of aftercare and support led many participants to perceive the assessments as a "waste of time" (R128, female, age 18-24, patient). Receiving out-of-date signposting information was frustrating for some people and others found the clinician's advice to be overly simplistic. Feeling unsupported during and after assessments led to despondency, hopelessness, and increased distress for some participants. In several instances, negative experiences of the assessment process and the lack of support led to further thoughts and acts of self-harm.

> "The assessment feels like, & is, a waste of time & makes you feel even more ashamed & worthless & more likely to self-harm again" (R21, female, age 45-49, patient).

For peer review only - http://bmjopen.bmj.com/site/about/guidelines.xhtml

<u>Help-seeking and engagement</u>

Experiences of attending the emergency department following self-harm had a cumulative effect and resulted in far-reaching consequences on help-seeking and engagement with mental health services for some participants. Some reported that the lack of a therapeutic assessment, inadequate follow-up care and/or long waiting lists to access support increased their levels of distress and disillusionment. Over a third of participants reported that they were less likely to seek help or engage with assessments and/or were more likely to leave the emergency department without an assessment.

> "I didn't feel like I was being treated as a very high priority, and went home feeling like there's no point in going to A&E when I'm suicidal from now on (even though that's what all health professionals seem to suggest)" (R15, male, age 18-24, patient).

Table 3: After the psychosocial assessment: Themes and exemplar quotations

**Themes**	
**Hope**	"before and during, I felt ashamed, like a fraud and saddened that I was putting my family and friends through all this, and even more so that I was now putting a drain on resources that could off been used for someone else who needed it more than I. After, I felt supported by family with me and the action plan we had all discussed. I felt there was a glimmer of hope in all the darkness" (R113, female, age 35-39 patient).
**Harm**	"I felt that there was no room to explore what was actually going on, that I was given little opportunity to express myself, and like the whole process was a bit of a tick-box exercise, rather than a supportive conversation or a way to come up with helpful suggestions. 9 times out of 10 these assessments left me feeling worse" (R101, female, age 30-34, patient).<br><br>"The RAID assessment made me feel listened to and gave me a next step. But the next step was pointless. I was back home feeling like nothing changed. So, I felt in many ways worse" (R70, female, age 25-29, patient).<br><br>"They tell you to simply go listen to music or have a bath and then discharge you and as a result, you attempt to end your life" (R30, female, age 18-24, patient).
**Help seeking and engagement**	" I felt judged whilst being asked questions about how I was feeling, and like I was being misunderstood and like I wasn't being taken seriously. And after the assessment and after discharge from A&E the vague advice I had been given made me even less likely to return to A&E in future" (R09, non-binary, age 18-24, patient).

For peer review only - http://bmjopen.bmj.com/site/about/guidelines.xhtml

DISCUSSION

Main findings

We found that the psychosocial assessment process was experienced as helpful on some occasions but harmful on others. Participants reported feeling better, less suicidal, and less likely to repeat self-harm after receiving compassionate care and collaborative assessments that focused on building a therapeutic relationship and providing help. Conversely, the pathway in the emergency department and the manner by which the assessment was conducted may have resulted in iatrogenic harm for some patients. Reports of substandard medical care and derogatory comments were common among participants, and compounded by what appear to be pre-conceived ideas among some staff members regarding service use patterns, self-harm repetition, and perceived attention-seeking behaviour. For some participants, cumulative experiences of long-waiting times, stigmatising attitudes, disjointed care, and overly standardised assessments contributed to heightened distress, disengagement, and in some cases further self-harm episodes.

Strengths and limitations

We conducted an online survey with non-probability sampling to recruit our participants, an approach that is subject to potential selection bias.[21] However, our aim was not to generalise to a broader population but to provide qualitative contextual experiences of attending the emergency department for psychosocial assessments. Self-harm is often stigmatised. Recruiting individuals who have harmed themselves can be challenging, especially for emergency department studies.[10-12,17] Our co-designed qualitative online survey enabled us to investigate a marginalised group of people to ensure that their experiences and perceptions of care are considered for service development. Over 100 people contributed their experiences of assessments and provided recommendations for improving practice. Participants were able to disclose their experiences in their own time, at their own discretion, and under their own control. Whilst the survey was designed with an emphasis on the generation of free text qualitative data, we were unable to probe responses or explore complex issues such as the

For peer review only - http://bmjopen.bmj.com/site/about/guidelines.xhtml

relationship between the socio-economic status of the patient and the quality of psychosocial assessments.

We have included a demographic profile of respondents to evaluate the relevance of our findings to clinical and carer groups. Our sample included a wide age range but only 17 (19.3%) of patients were aged 18 to 25 and five (5.7%) were over 60. Experiences were similar across age groups, but future research is needed to explore issues for younger and older adults when accessing services. We did not include patients under the age of 18 because of distinctness of service provision for this demographic group. This is an important area for further research, given the transition between adolescent and adult services.[22] Our sample is similar to Multicentre Monitoring Studies of Self-Harm in England[23] in terms of age, but we recruited more women, which is consistent with community surveys of self-harm.[10-12, 24] However, self-harm and suicide rates are rising amongst women.[25] Our results provide important information on the unmet needs for females attending the emergency department following self-harm. Our study included relatively small numbers of carers (13.7%), Black and minority ethnic groups (4%), and patients who were men (16%), which is similar to community studies of self-harm.[10, 24] Whilst experiences of assessments were consistent across sub-groups in our data, help-seeking and quality-of-care may vary considerably for some patients in these groups compared to White British females.

Only four participants had no self-reported psychiatric diagnosis (see Appendix 1) and many had been diagnosed with a personality disorder or complex post-traumatic stress disorder. Participants had all undergone psychosocial assessment, so may be more representative of patients with a greater level of need. Further research is needed to understand the needs of patients who present to hospital emergency departments and do not have a psychiatric diagnosis or history of mental health service use.

For peer review only - http://bmjopen.bmj.com/site/about/guidelines.xhtml

Qualitative surveys are a useful tool for gathering data on patient experiences.[14,16] However, there is potential to further exclude underrepresented populations in research, such as non-English speaking participants, or those with literacy issues, limited digital awareness or access, and people who do not wish to write about their experiences. Language around healthcare service use and help-seeking may also seem too abstract for some marginalised individuals. Further co-designed stand-alone studies and arts-led approaches specifically designed for these groups are indicated, to provide evidence for provision of more equitable access to services and better tailored treatments.

For peer review only

This is a qualitative study based on patients' experiences and their perceptions of care that they received. [sentences removed] Perceptions of care may be affected by previous negative experiences, poor follow-up, and psychological distress. Some individuals may perceive staff negatively and disengage with the assessment process if they have previously experienced stigmatising attitudes and/or have low self-worth.[12] Participants may also be more likely to participate in a study if they have negative experiences of services. However, negative experiences provide important opportunities for learning and improving services. Participants also provided examples of what they find helpful and recommendations for improving clinical practice; therefore, we can also learn from scenarios in which participants have reported positive experiences.

This is the largest qualitative study to have examined psychosocial assessment following self-harm presentations to the emergency department. Our study was strengthened by substantive patient and carer involvement throughout the research process. Analyses were conducted by a multidisciplinary team that included clinical and research expertise and people with lived experience. This enabled triangulation at a research and respondent level, which enhanced the robustness and validity of the findings.[26]

Our results provide rich and detailed information on the assessment process from a population at elevated risk of self-harm repetition and suicide.[1] We have addressed many limitations of previous

For peer review only - http://bmjopen.bmj.com/site/about/guidelines.xhtml

studies in this area (e.g., small sample sizes and lack of demographic information)[10] and the lack of patient/carer involvement. Our broad recruitment strategy included hospitals and community groups to gather a wide range of experiences from around the United Kingdom.

Comparisons with existing research

There appears to be little change in patient experiences of emergency departments after self-harm over the last 15 years.[10-12] Our findings are consistent with largely negative accounts of patients' experiences of 'hostile care' in emergency departments following self-harm.[27-34] For example, similar to our respondents, Pembroke[28] reported the harm arising from the lack of compassionate care in the emergency department:

> "The whole experience of Accident & Emergency is degrading for self-harmers. It just perpetuates the vicious circle. I am made to feel that I'm wasting their time and resources which results in me hating myself even more. This sustains my cutting. If only Accident & Emergency staff could realise that it would be easier for them and for us if they treated us humanely…" (Helen, p.23).[28]

The concerning incidents where people reported not being offered pain-relief for self-harm are consistent with what was found in other studies previously and patient reports.[10, 27, 32] Future studies should focus on the relationship between such incidents and patient safety following self-harm. Stigmatising attitudes of healthcare staff towards people who have harmed themselves and consequences for patients are well documented.[35-37] Similarly, our findings highlight pervasive negative attitudes towards people who have harmed themselves and also poor communication during the assessment process by some healthcare staff. They indicate that negative interactions with staff contributed and reinforced feelings of shame and internalised stigma. Further studies should explore the relationship between healthcare policies, cultures, systematic issues, and stigmatising attitudes towards people who have harmed themselves from a patient safety perspective.

Consistent with Hunter et al.,[11] we found that assessments could positively or negatively affect hopelessness and engagement with services. Patients reported uncertainty about the purpose of the assessment and their experiences with aftercare were often negative. Similar to Owens et al.[27] our

For peer review only - http://bmjopen.bmj.com/site/about/guidelines.xhtml

results highlight how the transition through the emergency department affects engagement, emotional states during and after the assessment, and future help-seeking. Similar to MacDonald and colleagues,[10] we found that transitions were apparently poorly managed for some participants, leading to uncertainty and feelings of abandonment by the end of the assessment.

Implications for clinical practice and policy

The novel findings that have been uncovered by this large qualitative study of patient/carer experiences of psychosocial assessment in the emergency department following self-harm suggest that elements of current practice are contrary to the NICE clinical recommendations[2] and national quality standards (QS34).[38] Greater public education on self-harm, clinical reflexivity, and good quality supervision may help to address some of the stigmatising attitudes in this area and improve patient experience. Table 3 compares the study's findings and their potential implications for practice.

Some of the poor experiences of care and assessment reported in this study possibly reflect a gap between current guidelines and their implementation, but others may reflect more fundamental challenges not just for guidelines but for clinical practice as a whole. For example, how do we balance the safety of service users with the provision of care that is as compassionate as it can be? What are the most appropriate outcome measures not just for self-harm research but for clinical services? The NICE guideline for the care of people who have harmed themselves are currently under revision and represent an opportunity to hopefully address some of these issues.

Measures defined by patients, other than risk of repeat self-harm and suicide may also help to improve patient experience.[39] To ensure cultural relevance, future co-designed studies should include the views of patients from different linguistic/ cultural backgrounds in policy developments (e.g., see Leckning and colleagues).[40] Further work is necessary to investigate clinician interpretation and implementation of guidelines in practice, alongside co-designed quality improvement initiatives which more closely evaluate patient experience in the emergency department.

For peer review only - http://bmjopen.bmj.com/site/about/guidelines.xhtml

Self-harm itself remains a policy priority, both as part of wider suicide prevention but also in its own right.[41] There needs to be a focus on research but also putting what we know into practice - assessments and evidence-based interventions should be available and accessible. Both staff training and service development must of course, be guided by those with lived experience.

[insert table 3 around here] .

Liaison psychiatry services have rapidly transformed to develop alternative models of care during the Covid-19 pandemic, including ward reconfigurations and diversions away from the emergency department.[8] Psychosocial assessments remain a core component of practice but the method and location of delivery may change (e.g., virtual/ telephone assessments). The Royal College of Psychiatrists recent survey of liaison psychiatry service changes during the covid-19 pandemic suggests that the stigmatisation of mental illness by acute hospital staff may increase during this time of change.[8] It is therefore imperative that we better understand and ameliorate the harmful impact of stigmatising attitudes, and that any service changes consider patient and carer experiences and perceptions of care.

For peer review only - http://bmjopen.bmj.com/site/about/guidelines.xhtml

Table 3 NICE quality standards for self-harm compared to the study results, and potential implications for practice

	NICE Quality standards [QS34]	Study results (N=102)	Potential implications for practice based on patient/carer recommendations
**1**	People who have harmed themselves are cared for with compassion and the same respect and dignity as any service user.	People experienced significant levels of stigmatising attitudes throughout the assessment process.	Given the levels of stigmatising attitudes reported by participants and elevated risk of suicide associated with self-harm and clinical populations (e.g., autism spectrum condition, obsessive compulsive disorder, post-traumatic stress disorder, personality disorders)[42] staff education that also considers reflexivity, culture, and socioeconomic factors may be helpful. Training may be more effective if tailored towards staff groups (e.g., acute staff, liaison psychiatry staff), co-designed and delivered by people with relevant lived experience.
**2**	People who have self-harmed have an initial assessment of physical health, mental state, safeguarding concerns, social circumstances, and risk of self-harm repetition or suicide.	Participants felt initial assessments were focused on their mental state without attention to their distress or reasons for self-harm.<br><br>Long waiting-times were compounded by the continued use of having to wait until the patient is 'medically cleared' prior to assessment	Emphatic care at initial stages may help to encourage people to stay for further assessment. Clear communication about the purpose of initial assessments, and roles of each staff member may clarify the process and expected outcomes.<br><br>Trained support workers or mental health volunteers could ease transitions by providing support, check-ins, and soothing aids where needed. Joint-working between acute and liaison staff from initial stages could help with patient flow, engagement, and experience.[43]
**3**	People who have self-harmed receive a comprehensive psychosocial assessment.	Participants were unsure of the purposes of the assessment. People hoped for help but often felt let down by the lack of therapeutic engagement and aftercare. The way the assessment was carried out affected how the person felt afterwards.	Clear communication about the process, purpose, and expectations for the assessment may help people to understand the process. Care and sensitivity could help people feel safer during this vulnerable stage. Collaborative assessments that focus on building a therapeutic relationship could engage people in the process and build up trust in mental health services.[44]

For peer review only - http://bmjopen.bmj.com/site/about/guidelines.xhtml

	NICE Quality standards [QS34]	Study results (N=102)	Potential implications for practice based on patient/carer recommendations
**4**	People who have self-harmed receive the monitoring that they need whilst in the healthcare setting, to reduce self-harm repetition risk.	Empathetic check-ins from staff or support workers helped to encourage people to stay for assessment and provide reassurance about the process. However, monitoring from security guards was experienced as perceived as coercive and affected engagement with the assessment process.	Our results suggest that the use of security guards to monitor patients may harmfully impact on levels of engagement and help-seeking. Other ways to check-in and monitor patients at risk may be helpful such as trained support workers and staff. Where detainment may be necessary; staff training in mental health, robust legal justification, and clear communication over the roles of the personnel involved may help to improve patient and staff experience.
**5**	People who have self-harmed are cared for in a safe physical environment while in the healthcare setting, in order to reduce self-harm repetition.	Participants described poor experiences of waiting for lengthy periods of time in unsafe healthcare environments.	Environments that are separate from the emergency department may be beneficial for physical and psychological safety. Having the opportunity to wait in separate environments or quite rooms could allow some recovery and distance from the noise and intensity of the emergency department, which may facilitate greater engagement.
**6**	People receive continuing support for self-harm have a discussion with their healthcare professional about the potential benefits of psychological interventions specifically structured for people who self-harm.	Follow-up care was a major source of disillusionment for participants. There was little discussion of psychological therapies. Long waiting times were common. Participants were desperate for help at the time of the assessment and for more therapeutic engagement.	Our results suggest that greater communication and transparency over psychological therapies and waiting times may be helpful. Therapeutic assessments and enhanced availability of psychological therapies delivered by the liaison psychiatry team may be beneficial for some patients and carers.
**7**	People receiving continuing support for self-harm and moving between mental health services have a collaboratively developed plan describing how support will be provided during the transition.	Poor transitions whilst in the emergency department and linking between services (e.g., primary-care, secondary-care) left many participants feeling distressed and abandoned. Some participants were unclear the role of care plans that had a directives for “do-not-assess.”	Our findings suggest that assessments should be offered for every hospital presenting self-harm episode and plans updated. Co-designed advance directives with accessible crisis may provide more control and understanding over assessment/treatment options for some patients.[44]<br><br>Given the challenges in accessing specialist services, emergency department presentations for self-harm provide an important

For peer review only - http://bmjopen.bmj.com/site/about/guidelines.xhtml

			opportunity for intervention at a time of crisis for the patient. Good quality, compassionate assessments, and collaboratively developed safety plans may help to ease acute distress and prevent repeat self-harm.[45] Training in clinician-patient communication may help to provide a shared understanding of patient issues, reduce miscommunication, and thereby enhance therapeutic engagement and quality-of-care. [46-47]

For peer review only

Statements:

Funding statement
This work was funded by the National Institute for Health Research (NIHR) Greater Manchester Patient Safety Translational Research Centre (Reference number: PSTRC-2016-003). The views expressed are those of the authors(s) and not necessarily those of the NIHR or the Department of Health and Social Care.

Competing interests
NK is a member of the Department of Health's (England) National Suicide Prevention Advisory Group. He chaired the NICE guideline development group for the longer-term management of self-harm and the NICE Topic Expert Group (which developed the quality standards for self-harm services). NK is currently chair of the updated NICE guideline for Depression and Topic Advisor to the new NICE self-harm guideline. He is also supported by the Greater Manchester Mental Health NHS Foundation Trust.

Acknowledgements:
We wish to thank the participants who have taken the time to generously share their often-painful experiences, and also our patient and carer panel (Dawn Allen, Stephen Barlow, David Daniels, Dan Stears, Elizabeth Monaghan, Paula Mazzucco, Manoj Mistry, Tracy Neil, Fiona Naylor, Javed Rehamn, Jonathan Smith), for their involvement and insights throughout the process. We are also grateful to our GM NIHR PSTRC staff stakeholder panel and to the FAST-R panel for their input into the study. We also wish to thanks staff at the mental health trusts and clinical research networks, as well as community organisations for facilitating the research and assisting with the recruitment. Finally, we wish to thank the reviewers for enabling us to improve the manuscript with their helpful suggestions.

Authorship & contribution
All authors made substantial contributions to the study. SC, RW, and NK were responsible for funding acquisition. We designed and developed the study through discussion at team meetings with LQ, DL, RW, NK, and at meetings with members of our patient/ public involvement (PPI) group. LQ coordinated data collection with assistance from DL. LQ and LG did the analyses with input from our wider PPI panel, and from EM, SB, DL, RW, and NK. LQ interpreted the results with input from LG, EM, SB, DL, RW, NK, and LQ wrote the first draft. All authors contributed to subsequent drafts and approved the final version. All authors take responsibility for the integrity of the data and accuracy of the data analysis. NK is the guarantor of the study.

Data availability
The data that support the findings of this study are not publicly available due to restrictions of the research (consent and information that could compromise the privacy of some research participants).

For peer review only

References

1. Carr M.J, Ashcroft DM, Kontopantelis E, While D, Awenat Y, Cooper J, et al. Premature death among primary care patients with a history of self-harm. Ann Fam Med. 2017a; 15(3): 246-254.
2. NICE. Self-harm. The NICE Guideline on Longer-term management. National Clinical Guideline Number 133. London: The British Psychological Society and The Royal College of Psychiatrists; 2011.
3. Olfson M, Wall M, Wang S, Crystal S, Gerhard T, & Blanco C. (2017). Suicide following deliberate self-harm. Am J Psychiatry. 2017; (8): 765-774.
4. Geulayov G, Casey D, McDonald KC, Foster P, Pritchard K, Wells C, et al. (2018). Incidence of suicide, hospital-presenting non-fatal self-harm, and community-occurring non-fatal self-harm in adolescents in England (the iceberg model of self-harm): a retrospective study. Lancet Psychiatry. 2018; 5(2): 167-174.
5. Department of Health (2019). Preventing Suicide in England: Fourth Progress Report of the Cross-Government Outcomes Strategy to Save Lives. London: HM Government.
6. Bergen H, Hawton K, Waters K, Cooper J, & Kapur N. Psychosocial assessment and repetition of self-harm: the significance of single and multiple repeat episode analyses. J Affect Disord 2010; 127(1): 257-265.
7. Steeg S, Emsley R, Carr M, Cooper J, & Kapur N. Routine hospital management of self-harm and risk of further self-harm: propensity score analysis using record-based cohort data. Psychol Med. 2018; 48(2): 315-326.
8. Royal College of Psychiatrists. Alternatives to emergency departments for mental health assessments during the COVID-19 pandemic. UK: Faculty of Liaison Psychiatry. 2020a

For peer review only

9. Holmes EA, O'Connor RC, Perry VH, Tracey I, Wessely S, Arseneault L et al. Multidisciplinary research priorities for the COVID-19 pandemic: a call for action for mental health science. Lancet Psychiatry. 2020; 7: 547-60.

10. MacDonald S, Sampson C, Turley R, Biddle L., Ring N, Begley R, et al. Patients' Experiences of Emergency Hospital Care Following Self-Harm: Systematic Review and Thematic Synthesis of Qualitative Research. Qual Health Res. 2020; 30(3): 471-485.

11. Hunter C, Chantler K, Kapur N, & Cooper J. Service user perspectives on psychosocial assessment following self-harm and its impact on further help-seeking: a qualitative study. J Affect Disord. 2013; 145(3): 315-323.

12. Horrocks J, Hughes J, Martin C, House A, & Owens D. Patient experiences of hospital care following self- harm—A qualitative study. UK: University of Leeds. 2005.

13. Teddlie, C., & Tashakkori, (2015). Overview of contemporary issues in mixed methods research. In SAGE Handbook of Mixed Methods in Social and Behavioural Research. Thousand Oaks: SAGE Publications Inc.

14. Braun V, Clarke V, Boulton, E, Davey L, McEvoy C. (2020). The online survey as a qualitative research tool. Int J Soc Res Methodol [internet] 2020 cited 17 August 20] Available from: https://www-tandfonline-com.manchester.idm.oclc.org/doi/full/10.1080/13645579.2020.1805550

15. Kaushik V, Walsh CA. Pragmatism as a research paradigm and its implications for social work research. Social Sciences. 2019;8(9):255

16. Gibson, S., Boden, Z. V., Benson, O., & Brand, S. L. (2014). The impact of participating in suicide research online. *Suicide and Life-Threatening Behavior*, *44*(4), 372–383. doi:10.1111/sltb.12082

17. Kapur N, Gunnell D, Hawton K, Nadeem S, Khalil S, Longson D, et al. Messages from Manchester: pilot randomised controlled trial following self-harm. BJ Psych. 2013; 203(1): 73-74.

For peer review only

18. Fereday J, & Muir-Cochrane E. Demonstrating rigor using thematic analysis: A hybrid approach of inductive and deductive coding and theme development. Int J Qual Methods. 2006: 5(1): 80–92. doi: 10.1177/160940690600500107

19. IBM Corp. Released 2013. IBM SPSS Statistics for Windows, Version 22.0. Armonk, NY: IBM Corp.

20. QSR International Pty Ltd. NVivo (Version 12), https://www.qsrinternational.com/nvivo-qualitative-data-analysis-software/home NVivo qualitative data analysis software; QSR International Pty Ltd. Version 11, 2015.

21. Pierce M, McManus S, Jessop C, John A, Hotopf M, Ford T, et al. Says who? The significance of sampling in mental health surveys during COVID-19. Lancet Psychiatry. 2020; 7, 7: 567-568.

22. Singh SP, Paul M, Ford T, Kramer T, Weaver T, McLaren, S, et al. Process, outcome and experience of transition from child to adult mental healthcare: multiperspective study. BJ Psych. 2010; 197(4): 305-312.

23. Geulayov G, Kapur N, Turnbull P, Clements C, Waters K, Ness J, Townsend E, Hawton K. Epidemiology and trends in non-fatal self-harm in three centres in England, 2000–2012: findings from the Multicentre Study of Self-harm in England. BMJ Open. 2016 Apr 1;6(4).

24. Samaritans. Pushed from pillar to post. UK. Samaritans. 2020.

25. Department of Health and Social Care. The Women's Mental Health Taskforce. Final Report. UK: Department of Health. 2018.

26. Peters S. Qualitative Research Methods in Mental Health. Evid Based Ment Health. 2010; 13: 35-40.

27. Owens C, Hansford L, Sharkey S, & Ford T. "Needs and fears of young people presenting at accident and emergency department following an act of self-harm: secondary analysis of qualitative data". BJ Psych. 2016; 208: 286-291.

28. Pembroke. Self-harm: perspectives from personal experience, Survivors Speak Out. London. 1994.

For peer review only - http://bmjopen.bmj.com/site/about/guidelines.xhtml

29. Taylor TL, Hawton K, Fortune S, Kapur N. Attitudes towards clinical services among people who self-harm: systematic review. BJ Psych. 2009 Feb;194(2):104-10.

30. Chandler, A. Self-injury, medicine, and society: Authentic bodies. Palgrave Macmillan. 2016.

31. Hadfield J, Brown D, Pembroke L, Hayward M. Analysis of accident and emergency doctors' responses to treating people who self-harm. Qual Health Res. 2009;19(6):755-65.

32. DrEm_79. (2016). What your patient is thinking: Self-harm in the emergency department. 2016 *BMJ*, 353: 1150.

33. Chandler, A. (2018). Seeking secrecy: A qualitative study of younger adolescents' accounts of self-harm. *Young*, *26*(4), 313-331.

34. Harris J. Self-harm: Cutting the bad out of me. Qual Health Res. 2000;10(2):164-73.

35. Karman P, Kool N, Poslawsky IE, van Meijel B. Nurses' attitudes towards self-harm: a literature review. J Psychiatr Ment Health Nurs. 2015;22(1):65-75.

36. Rayner G, Blackburn J, Edward KL, Stephenson J, Ousey K. Emergency department nurse's attitudes towards patients who self-harm: A meta-analysis. Int J Ment Health Nurs. 2019;28(1):40-53.

37. Saunders KE, Hawton K, Fortune S, Farrell S. Attitudes and knowledge of clinical staff regarding people who self-harm: a systematic review. J Affect Disord Rep. 20121;139(3):205-16.

38. NICE. Self-harm Quality Standard (QS34). UK. National Institute for Health Research. 2013. https://www.nice.org.uk/guidance/qs34/resources/selfharm-pdf-2098606243525

39. Allen S. Self-harm and the words that bind: a critique of common perspectives. J Ment Heath Health Nurs. 2007;14(2):172-8.

40. Leckning B, Hirvonen T, Armstrong G, Carey TA, Westby M, Ringbauer A, Robinson G. Developing best practice guidelines for the psychosocial assessment of Aboriginal and Torres Strait Islander people presenting to hospital with self-harm and suicidal thoughts. Aust N Z J Psychiatry. 2020 Sep;54(9):874-82.

For peer review only - http://bmjopen.bmj.com/site/about/guidelines.xhtml

41. Kapur N. Services for self-harm: progress and promise? BJ Psych. 2020 Dec;217(6):663-4.

42. Ropper A H, Fazel S, & Runeson B. Suicide. N Eng J Med. 2020; 38: 266-74.

43. Brown S, Eales S, Hayhurst C, Hood S, McAllister E, Potts S, & Thomson, A. Side by Side: A UK-wide consensus statement on working together to help patients with mental health needs in acute hospitals. UK: Royal College of Psychiatrists. 2020. https://www.rcpsych.ac.uk/docs/default-source/members/faculties/liaison-psychiatry/liaison-sidebyside.pdf

44. Palombini E, Richardson J, McAllister E, Veale D, & Thompson AB. (2020). When self-harm is about preventing harm: emergency department management of obsessive-compulsive disorder and associated harm. BJPsych Bull [internet]. 2020 [cited 2nd July]. Available from https://doi-org.manchester.idm.oclc.org/10.1192/bjb.2020.70.

45. Royal College of Psychiatrists. CR229: Self-harm and suicide in adults. Final report of the Patient Safety Group. UK: Royal College of Psychiatrists. 2020b. Available from: https://www.rcpsych.ac.uk/docs/default-source/improving-care/better-mh-policy/college-reports/cr229_self-harm-and-suicide.pdf

46. McCabe R, & Healey PG. Miscommunication in doctor–patient communication. Top Cogn Sci. 2018; 10(2), 409-424.

47. Parker D, Byng R, Dickens C, Kinsey D, & McCabe R. Barriers and facilitators to GP–patient communication about emotional concerns in UK primary care: a systematic review. Fam Pract. 2020: 1-11.

For peer review only - http://bmjopen.bmj.com/site/about/guidelines.xhtml

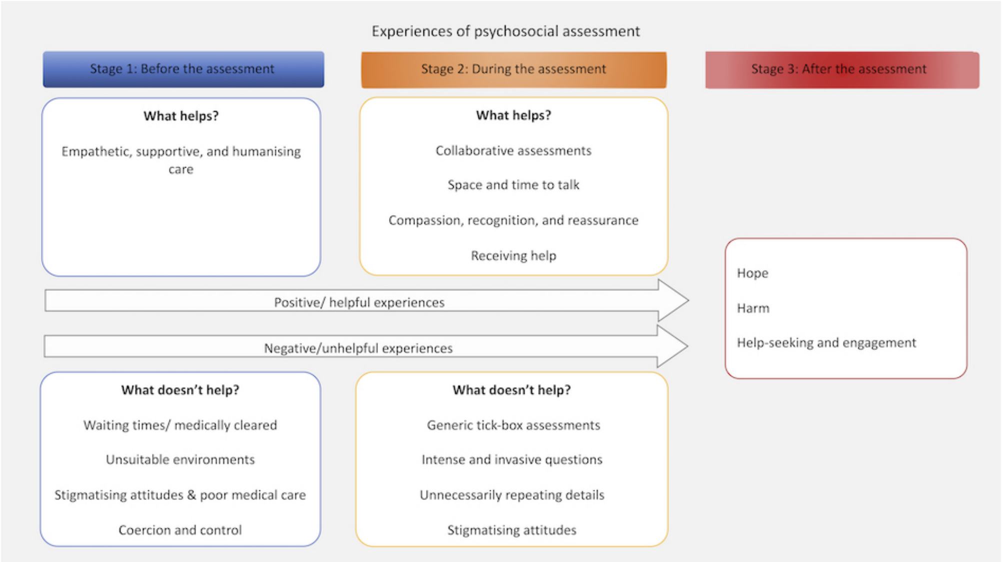

Figure 1. Themes and sub-themes across the three assessment process stages (1. Before the assessment; 2. During the assessment; 3. After the assessment)

80x44mm (300 x 300 DPI)

Appendix 1. Additional methodological details

Recruitment

This study was conducted as part of a larger programme of applied research investigating psychosocial assessments and psychological therapies following self-harm in the NIHR Greater Manchester Patient Safety Translational Research Centre. The survey was accessed through a University of Manchester website that contained a summary of the research process, a link to the participants' information sheet, consent form, and post-assessment debrief statement. We expected to recruit approximately 100 respondents, an estimate that was based on our previous studies that were conducted using online surveys.[2-3]

Online survey procedure

The study website, online survey tool, question format (e.g., yes/no, free-text; how the questions were asked), and questions were devised collaboratively with our patient/carer advisory panel via a series of workshops. We included open and closed text questions (e.g., demographics, selecting choices for services; binary response experiences) to elicit participant's views and experiences of assessments and follow-up care (see Box 1). We also asked for self-reported diagnosis, age, gender, education, and where the geographic area where the person resided. Survey questions for patients and carers were similar. Carers were invited to provide proxy information for diagnosis, living arrangements, and employment; and share their views of assessments for the person presenting with self-harm. Participants could tick boxes, skip questions, and/or complete free text responses without word limits. All participants provided informed consent for their participation. Due to the sensitive nature of the topic, links were provided to several online support sites after the assessment. Informed consent was obtained from all participants which was indicated by clicking continue on the survey.

For peer review only

Box 1. Survey questions

- What is your experience of mental health assessments after you have presented to the emergency department with self-harm? Please tell us what worked well. Please tell us what worked less well.
- How did you feel about having the mental health assessment? How did you feel during the assessment? How did you feel after the assessment?
- Can you think of any reasons when you would be more likely to receive an assessment in the emergency department after self-harm?
- Can you think of any reasons when you would be less likely to receive an assessment following self-harm?
- Have you ever not received or been refused a mental health assessment following self-harm?
- Have you ever refused a mental health assessment?
- Have you ever left the emergency department before being assessed by a mental health clinician?
- Is there anything that would make it more likely that you would accept or stay for a mental health assessment?
- How would you change or do things differently?

Data were analysed and interpreted by a multidisciplinary team that included expertise in psychiatry, mental health services research, psychology, and qualitative methodologies. The public contributors involved in the analyses included people with lived experience of self-harm and attending mental health services after harming themselves, as well as carers of these individuals.

Participant demographics

The majority of patients were residing in England (99/102, 97.1%) and were of White British or Irish ethnicity (92/102, 91.1%). Most patients were either in paid or voluntary work (41/102, 41.4%), or were receiving statutory disability benefits (39/102, 39.4%); 9/102 (8.8%) were students and the remainder were not working (10/102, 10.1%). Many patients had a self-reported psychiatric diagnosis

For peer review only - http://bmjopen.bmj.com/site/about/guidelines.xhtml

including the following disorders (that are not mutually exclusive): depression (60/102, 58.8%), emotionally unstable personality disorder (43/102, 42.2%), anxiety disorders (37/102, 36.3%), post-traumatic stress-disorder (25/102, 24.5%), eating disorders (13/102, 12.7%), psychotic disorders (10/102, 9.8%), bipolar disorder (10/102, 9.8%), autism spectrum disorder (7/102, 6.9%), obsessive compulsive disorder (6/102, 5.9%) dissociative disorders (4/102, 3.9%), and dysmorphic disorders (1/102, 1.0%).

References

1. Littlewood, D. L., Quinlivan, L., Steeg, S., Bennett, C., Bickley, H., Rodway, C., ... & Kapur, N. (2019). Evaluating the impact of patient and carer involvement in suicide and self-harm research: A mixed-methods, longitudinal study protocol. *Health Expectations*, 00:1-7.
2. Quinlivan, L., Nowland, R., Steeg, S., Cooper, J., Meehan, D., Godfrey, J., ... & Allen, N. (2019). Advance decisions to refuse treatment and suicidal behaviour in emergency care: 'it's very much a step into the unknown'. *BJPsych open*, *5*(4).
3. Flynn, S., Raphael, J., Graney, J., Nyathi, T., Williams, A., Kapur, N., ... & Shaw, J. (2019). The personality disorder patient pathway: Service user and clinical perspectives. *Personality and mental health*, *13*(3), 134-143.

For peer review only - http://bmjopen.bmj.com/site/about/guidelines.xhtml

Standards for Reporting Qualitative Research (SRQR)

O'Brien B.C., Harris, I.B., Beckman, T.J., Reed, D.A., & Cook, D.A. (2014). Standards for reporting qualitative research: a synthesis of recommendations. *Academic Medicine, 89(9)*, 1245-1251.

No. Topic	Item	Page number
**Title and abstract**		
S1 Title	Concise description of the nature and topic of the study identifying the study as qualitative or indicating the approach (e.g., ethnography, grounded theory) or data collection methods (e.g., interview, focus group) is recommended	Page 1
S2 Abstract	Summary of key elements of the study using the abstract format of the intended publication; typically includes objective, methods, results, and conclusions	Page 2
**Introduction**		
S3 Problem formulation	Description and significance of the problem/phenomenon studied; review of relevant theory and empirical work; problem statement	Page 4
S4 Purpose or research question	Purpose of the study and specific objectives or questions	Page 4-5
**Methods**		
S5 Qualitative approach and research paradigm	Qualitative approach (e.g., ethnography, grounded theory, case study, phenomenology, narrative research) and guiding theory if appropriate; identifying the research paradigm (e.g., positivist, constructivist/interpretivist) is also recommended	Page 6
S6 Researcher characteristics and reflexivity	Researchers' characteristics that may influence the research, including personal attributes, qualifications/experience, relationship with participants, assumptions, or presuppositions; potential or actual interaction between researchers' characteristics and the research	Appendix 2, page 3

For peer review only

	questions, approach, methods, results, or transferability	
S7 Context	Setting/site and salient contextual factors; rationale[a]	Page 6
S8 Sampling strategy	How and why research participants, documents, or events were selected; criteria for deciding when no further sampling was necessary (e.g., sampling saturation); rationale[a]	Page 6
S9 Ethical issues pertaining to human subjects	Documentation of approval by an appropriate ethics review board and participant consent, or explanation for lack thereof; other confidentiality and data security issues	Page 6 and in Appendix 2.
S10 Data collection methods	Types of data collected; details of data collection procedures including (as appropriate) start and stop dates of data collection and analysis, iterative process, triangulation of sources/methods, and modification of procedures in response to evolving study findings; rationale[a]	Page 6, 7 and in Appendix 2.
S11 Data collection instruments and technologies	Description of instruments (e.g., interview guides, questionnaires) and devices (e.g., audio recorders) used for data collection; if/how the instrument(s) changed over the course of the study	Page 6 and Appendix 2.
S12 Units of study	Number and relevant characteristics of participants, documents, or events included in the study; level of participation (could be reported in results)	Page 8. Additional information is presented in Appendix 2.
S13 Data processing	Methods for processing data prior to and during analysis, including transcription, data entry, data management and security, verification of data integrity, data coding, and anonymization/deidentification of excerpts	Page 7 and Appendix 2.
S14 Data analysis	Process by which inferences, themes, etc., were identified and developed, including researchers involved in data analysis; usually references a specific paradigm or approach; rationale[a]	Page 7 and Appendix 2.
S15 Techniques to enhance trustworthiness	Techniques to enhance trustworthiness and credibility of data analysis (e.g., member checking, audit trail, triangulation); rationale[a]	Page 4, page 7, Appendix 2, and the discussion on page 19.
**Results/Findings**		
S16 Synthesis and interpretation	Main findings (e.g., interpretations, inferences, and themes); might include development of a theory or model, or integration with prior research or theory	
S17 Links to empirical data	Evidence (e.g., quotes, field notes, text	

	excerpts, photographs) to substantiate analytic findings	
**Discussion**		
S18 Integration with prior work, implications, transferability, and contribution(s) to the field	Short summary of main findings; explanation of how findings and conclusions connect to, support, elaborate on, or challenge conclusions of earlier scholarship; discussion of scope of application/generalizability; identification of unique contribution(s) to scholarship in a discipline or field	Page 17
S19 Limitations	Trustworthiness and limitations of findings	Page 17
**Other**		
S20 Conflicts of interest	Potential sources of influence or perceived influence on study conduct and conclusions; how these were managed	In the statements section.
S21 Funding	Sources of funding and other support; role of funders in data collection, interpretation, and reporting	In the statements section.

[a]The rationale should briefly discuss the justification for choosing that theory, approach, method, or technique rather than other options available, the assumptions and limitations implicit in those choices, and how those choices influence study conclusions and transferability. As appropriate, the rationale for several items might be discussed together.

For peer review only
